# Formate- and Sodium Ion-Dependent Wood–Ljungdahl Pathway Involved in Organo‑, Litho‑, and Methylotrophic Growth of *Tepidanaerobacter acetatoxydans*

**DOI:** 10.1021/acs.est.6c09717

**Published:** 2026-08-25

**Authors:** Raphael Trischler, Lennox J.-V. Fletcher, Volker Müller

**Affiliations:** Molecular Microbiology & Bioenergetics, Institute of Molecular Biosciences, Johann Wolfgang Goethe University, Max-von-Laue Str. 9, Frankfurt D-60438, Germany

**Keywords:** Tepidanaerobacter acetatoxydans, formate dehydrogenase, Wood–Ljungdahl pathway, methylotrophic growth

## Abstract

Methane production by a biogas digester is a promising alternative to the use of fossil fuels as an energy source. One major substrate for methanogenesis is acetate, which is directly used by acetoclastic methanogens. At high pH and high ammonium concentrations, acetoclastic methanogenesis is inhibited, and acetate is oxidized to H_2_ + CO_2_, which are used by hydrogenotrophic methanogens. From such a digester, an ammonium-tolerant syntrophic acetate-oxidizing bacterium, *Tepidanaerobacter acetatoxydans,* was isolated in 2011 by Westerholm and colleagues, but its physiology and role in a biogas digester are still relatively unknown. We demonstrate that *T. acetatoxydans* is an acetogenic bacterium using an unusual Wood–Ljungdahl pathway (WLP) lacking a formate dehydrogenase. Therefore, *T. acetatoxydans* cannot ferment glucose, H_2_ + CO_2_, or CO + CO_2_, but the addition of formate restored acetogenesis. *T. acetatoxydans* could also grow on methyl groups that were oxidized in the WLP, but due to the absence of a formate dehydrogenase, methyl groups were incompletely oxidized to formate instead of CO_2_ as an end product. This study gives new insights into the physiology of acetogens that lack formate dehydrogenases and sheds new light on the role of *T. acetatoxydans* in the food web of a biogas digester.

## Graphical abstract

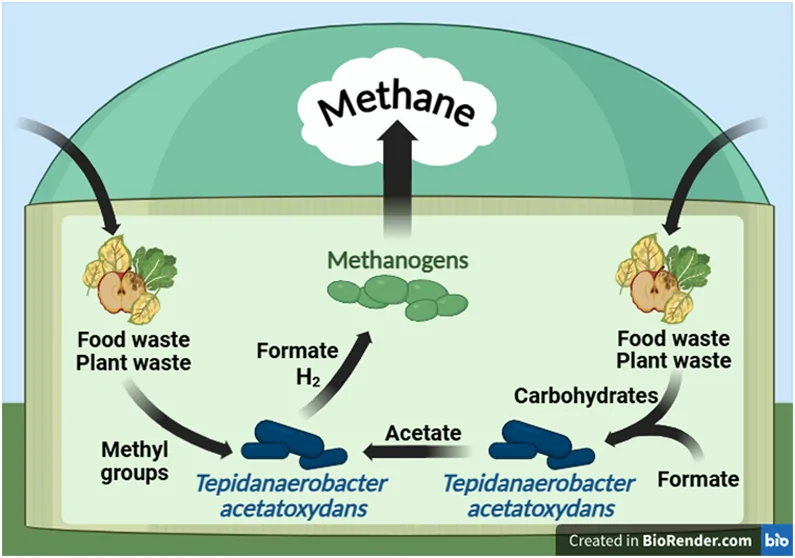

## Introduction

In times of climate change, the search for novel and renewable energy sources is one of the biggest challenges that society has to face. An alternative to fossil fuels are biogas digesters that produce the energy carrier methane from organic waste products. In a biogas digester, complex organic matter is first hydrolyzed by primary degraders. The resulting monomers are then further fermented to alcohols, acids, H_2_, and CO_2_, compounds that can be cross-fed to methanogenic archaea and acetogenic bacteria.^1,2^ Methanogens are the catalysts for methane production and commonly use H_2_ + CO_2_ as substrates; some can use methyl groups, and a few use acetate.^2–4^ Acetogens, on the other hand, have a much broader substrate spectrum; they can also use C1 substrates (H_2_ + CO_2_, methyl groups, formate, CO) but, in addition, a wide range of different organic substrates.^5^ The main fermentation end product of hexoses in acetogens is acetic acid. Two mol of acetate are formed by way of glycolysis, and the electrons gained from glycolysis and pyruvate oxidation are channeled into the Wood–Ljungdahl pathway (WLP) to reduce 2 mol of CO_2_ to acetate. Thus, hexose fermentation is homoacetogenic.^6^ The WLP is a linear, two-branched pathway in which two molecules of CO_2_ are reduced to an acetyl moiety and condensed with CoA to give acetyl-CoA. Acetate is formed via acetyl phosphate. In fact, acetogenic bacteria are categorized by the ability to use this pathway.^6–10^ In the methyl branch, CO_2_ is first reduced to formate, which is then bound to tetrahydrofolate and reduced to a methyl group.^11–13^ In the carbonyl branch, another CO_2_ is reduced to CO and bound to the key enzyme of the pathway, CO dehydrogenase/acetyl-CoA synthase complex (CODH/ACS), that catalyzes acetyl-CoA formation.^14–16^

If biomass digesters contain plant material as feedstock, then methoxylated compounds present in pectin and lignin are also used as carbon and energy sources by acetogens; they are hydrolyzed to free methanol.^17^ Methyl groups from O-, N-, and S-methyl compounds, including aromatic substrates, are transferred into the WLP by action of methyltransferases: a methyltransferase I (MTI) abstracts the methyl group from the substrate and transfers it to a corrinoid protein (CoP), and methyltransferase II (MTII) transfers the methyl group to tetrahydrofolic acid (THF), giving methyl-THF.^18,19^ The fate of the methyl groups (production of acetate and oxidation to H_2_ + CO_2_) depends on the environmental conditions.^19^

Acetate is the central intermediate in the food web of a biogas digester and is a precursor of methane. Methane can be derived via two routes from acetate.^20^ While acetoclastic methanogens directly split acetate into a methyl and a carbonyl group to produce methane and CO_2_, the mechanism of syntrophic acetate oxidation (SAO) involves two steps, catalyzed by two different groups of organisms.^20^ The first group oxidizes acetate to CO_2_ and H_2_, and the second group contains hydrogenotrophic methanogens. Acetate is activated to acetyl-CoA, which is then split by CODH/ACS, and the methyl group and CO are oxidized to CO_2_ by a reversal of the WLP. Thus, syntrophic acetate oxidizers use the WLP in reverse. The delicate balance between two acetate oxidation pathways can be unbalanced when high-protein waste products are fed to the digester. Fermentation of proteins releases ammonium and leads to an increase in the pH of the digester. High ammonium and high pH inhibit growth of aceticlastic methanogens, and syntrophic acetate oxidation becomes the predominant pathway for methane formation.^21–24^ From such a digester, *Tepdianaerobacter acetatoxydans* was isolated in 2011; this bacterium has the highest ammonia tolerance (up to 1 M) reported so far.^25^ *T. acetatoxydans* was reported to grow on glucose; final yields were not reported, but growth was very poor.^25^ Acetate was the only product formed, which is in line with the hypothesis that *T. acetatoxydans* is an acetogenic bacterium. This notion was strengthened by the finding of all WLP genes, except for a formate dehydrogenase (Fdh) encoding gene. A co-culture with the hydrogenotrophic methanogen *Methanoculleus* sp. strain MAB2 resulted in acetate degradation and methane production,^25^ demonstrating the acetate-oxidizing capability of *T. acetatoxydans*, but again provides no proof for the operation of the WLP. In this study, we demonstrated an active WLP in *T. acetatoxydans*. Furthermore, this study elucidates the physiology of Fdh-lacking acetogens and gives further insight into the role of acetogens in the complex food web of a biogas digester.

## Material and Methods

### Organism and Cultivation

*T. acetatoxydans* Re1 (DSM 21804) was grown in CO_2_/KHCO_3_-buffered complex medium,^26^ including 2 g/L yeast extract as well as in NaCl-limited medium^27^ at 45 °C. Twenty mM glucose and different formate concentrations ranging from 5 to 400 mM were used as carbon and energy sources. In addition, *T. acetatoxydans* was cultivated with 5 mM syringate. Growth was recorded by measuring the optical density at 600 nm (OD_600_) in Hungate tubes (5 mL medium, 10 mL gas phase) or serum flasks (50 mL medium, 65 mL gas phase). This study used the strain *T. acetatoxydans* Re1 (DSM 21804); no live animals or human participants were involved, and no ethics approval was required.

### Preparation of Resting Cells

*T. acetatoxydans* was grown with 20 mM glucose and 20 mM formate as substrates or with 5 mM syringate as substrate in CO_2_/KHCO_3_-buffered complex medium at 45 °C to the late-exponential growth phase. Cells were harvested by centrifugation (8000*g*, 4 °C, 10 min, AvatiTM JA-10 Fixed-Angle Rotor, Beckman Coulter, Brea, CA, USA), washed, and resuspended in imidazole buffer (50 mM imidazole-HCl, 20 mM MgSO_4_, 20 mM KCl, 2 mM 1,4- dithioerythritol (DTE), 4.4 μM resazurin, pH 7.0). The final protein concentration was measured by the method of Schmidt et al. (1963).^28^ Resting cells were prepared in an anaerobic chamber (Coy Laboratory Products, Grass Lake, MI, USA) with an atmosphere of N_2_/H_2_ [96–98%: 2–4% (v/v)] under strictly anoxic conditions.

### Cell Suspension Experiments

Resting cells were resuspended to total protein concentration of 1 mg/mL in a final volume of 10 mL imidazole buffer (50 mM imidazole, 20 mM KCl, 20 mM MgSO_4_, 60 mM KHCO_3_, 2 mM DTE, 4.4 μM resazurin, pH 7.0) with or without 20 mM NaCl under an atmosphere of N_2_/CO_2_ [80:20 (v/v)] in 115 mL serum flasks. As substrates, 5 mM glucose, 10 mM formate, 5 mM syringate, as well as the gases H_2_ + CO_2_ (1 bar, [80:20 (v/v)]) and CO (20%) were used. Additionally, resting cells were incubated in the presence of the protonophore 3,3′,4′,5-tetrachlorosalicylanilide (TCS; 30 μM; 99%, Thermo Scientific Chemicals, Dreieich, Germany) or the Na^+^-ionophore ETH2120 (30 μM; Sodium ionophore III, ≥ 98.0%, Sigma-Aldrich, Darmstadt, Germany).

### Metabolite Analysis

The metabolites acetate^29^ and H_2_ ^30^ were determined by gas chromatography. The concentrations of formate and glucose were measured enzymatically using the “Enzytec Liquid Formic acid”-kit and “Enzytec (lowest detection limit 0.01 mM) Liquid d-Glucose”-kit from R-Biopharm AG (Darmstadt, Germany). Syringate was measured by high-performance liquid chromatography as described.^31^ For separation, a Poroshell 120 EC-C18 column (3 × 150 mm, 2.7 μM) (Agilent Technologies, Santa Clara, CA, USA), with a mixture of MQ/methanol/formic acid [80:20:0.16 (v/v/v)] as eluent (flow rate 0.5 mL/min; oven temperature 40 °C), was used. Five microliters of the sample were injected by the autosampler and analyzed by a 1260 Infinity II Diode Array Detector (Agilent Technologies, Santa Clara, CA, USA) operating at 272 nm.

### Measurement of Na^+^ Concentrations

For the determination of the concentration of sodium ions, a Na^+^-specific electrode (Orion Star A214; Thermo Scientific, MA, USA; detection limit for sodium ions: 5 μM) was used.^32^ The calibration curve was recorded using NaCl concentrations of 0.0, 0.01, 0.1, 1, 10, 100, and 1000 mM. To prepare buffers with reduced Na^+^ concentrations, ultrapure chemicals with low Na^+^ contamination (≤0.005%; Sigma-Aldrich, St. Louis, USA; Carl Roth GmbH + Co. KG, Karlsruhe, Germany) were used.^32^

### Preparation of Cell-Free Extract

*T. acetatoxydans* was cultivated in KHCO_3_/CO_2_-buffered complex medium (2 L) with 20 mM glucose and 20 mM formate as substrate at 45 °C to the late exponential growth phase (OD_600_ = 1.5). Cells were harvested by centrifugation (8000*g*, 4 °C, 10 min, AvatiTM JA-10 Fixed-Angle Rotor, Beckman Coulter, Brea, CA, USA), washed, and resuspended in 7 mL lysis buffer (50 mM Tris, 20 mM MgSO_4_, 2 mM DTE, 4.4 μM resazurin, pH 7.5) with 0.5 mM phenylmethylsulfonyl fluoride (PMSF) and 0.1 mg/mL DNase I. Afterwards, cells were disrupted by passing through a French pressure cell at 100 MPa. Finally, cell debris was removed by centrifugation (25000*g*, 30 min; Centrifuge 5417R, Eppendorf, Hamburg-Eppendorf, Germany). All steps were performed in an anaerobic chamber (Coy Laboratory Products, Grass Lake, MI, USA) within an atmosphere of N_2_/H_2_ [96–98%: 2–4% (v/v)]. The protein concentration was determined as described by Bradford (1976).^33^

### Determination of Enzyme Activities in Cell-Free Extract

Enzyme assays were performed under strictly anoxic conditions with glass cuvettes (*d* = 0.5 cm; Glasgerätebau Ochs, Germany) in Tris buffer (100 mM Tris, 2 mM DTE, 4.4 μM resazurin, pH 7) at 45 °C. Methylene-THF dehydrogenase was measured by reduction of 1 mM NADP^+^ at 340 nm with methylene-THF as electron donor, synthesized from 1.5 mM formaldehyde and 0.5 mM THF.^34^ Formate dehydrogenase, CO dehydrogenase as well as hydrogenase activities were determined by the reduction of 10 mM methylviologen or benzylviologen at 604 nm with 20 mM formate, CO (1 bar, 100%) or H_2_ (1 bar, 100%) as electron donor as described.^35^ Pyruvate:ferredoxin oxidoreductase (PFOR) and pyruvate:formate lyase (PFL) activities were determined as described.^36^ For the determination of the PFOR activity, the reduction of ferredoxin (isolated from *Clostridium pasteurianum* ^37^), in the presence of CoA, thiaminpyrophosphate, and pyruvate as electron donors, was measured. PFL activity was measured by the production of formate from pyruvate in the presence of CoA, using the “Enzytec Liquid Formic acid”-kit from R-biopharm AG (Darmstadt, Germany).

## Results

### Metabolic Features of *T. acetatoxydans* Derived from the Genome Sequence

The genome of *T. acetatoxydans* was fully sequenced by Müller et al. (2015),^38^ revealing the presence of WLP-encoding genes but absence of a formate dehydrogenase-encoding gene. In addition, genes encoding an Rnf complex, an electron-bifurcating hydrogenase, and transhydrogenase were identified. To further elucidate the physiological capacities of *T. acetatoxydans*, we analyzed the genetic organization of WLP-encoding genes and genes encoding energy-converting complexes in more detail. In sharp contrast to the acetogen *Acetobacterium woodii*, the WLP-encoding genes are not located in three, but in one gene cluster (Figure 1). This gene cluster harbors all genes coding for the CODH/ACS complex as well as all genes coding for the methyl branch of the WLP, except for a formate dehydrogenase. The methylene-THF reductase (MTHFR) of *T. acetatoxydans* is encoded by the genes *metV* and *metF*, indicating a ferredoxin-dependent Type II MTHFR.^39^ Interestingly, the genome of *T. acetatoxydans* harbors a second gene cluster encoding a second copy of each methyl branch encoding enzyme (without a formate dehydrogenase-encoding gene) (Figure 1). The corresponding gene products of both clusters were not identical (comparison of amino acid identities of WLP enzymes encoded by cluster I and cluster II: *fhs1*, 74.01%; *fchA*, 54.07%; *folD*, 53.87%; *metV*, 52.17%; *metF*, 57.93%), indicating that the presence of the second WLP-encoding gene cluster in the genome of *T. acetatoxydans* might not be a result of gene duplication but of horizontal gene transfer. Comparison of the amino acid sequences of the gene products of these two gene clusters with the corresponding gene products of *A. woodii* revealed amino acid identities ranging from 52 to 74% (Figures 1 and S1–S5). Interestingly, cluster II is in direct proximity to genes potentially encoding several methyltransferase systems (Figure 1). This cluster encodes six MTI, four CoP, and one MTII (Figure 1). Additional methyltransferase-encoding genes were not found elsewhere in the genome. Notably, three MTI-encoding genes are each followed by a transporter-encoding gene, which might enable the transport of methylated compounds into the cell. Additionally, the genome of *T. acetatoxydans* harbors genes encoding a glycine synthase pathway; two of the genes are directly upstream of *metF* (Figure 1). The glycine synthase pathway is a second pathway for CO_2_ fixation, in addition to the WLP. This pathway contains a H protein (*gcvH1*, TEPIRE1_3440; *gcvH2*, TEPIRE1_3870; *gcvH3*, TEPIRE1_6260), a dihydrolipoamide dehydrogenase (*lpdA1*, TEPIRE1_3430; *lpdA2*, TEPIRE1_6200; *lpdA3*, TEPIRE1_7170), an aminomethyltransferase (*gcvT1*, TEPIRE1_38600; *gcvT2*, TEPIRE1_24280), and a glycine dehydrogenase (*gcvPA*, TEPIRE1_3880; *gcvPB*, TEPIRE1_3890), which are encoded multiple times in the genome of *T. acetatoxydans*. Similar to *A. woodii* or *Clostridium drakei*,^40,41^ these genes are not clustered in the genome of *T. acetatoxydans*. In this pathway, the WLP-intermediate methylene-THF is condensed with CO_2_ to form glycine, which can be further metabolized to serine and pyruvate. The glycine synthase pathway enzymes of *T. acetatoxydans* are 39 to 63%, similar to the *A. woodii* enzymes.

**Figure 1:**
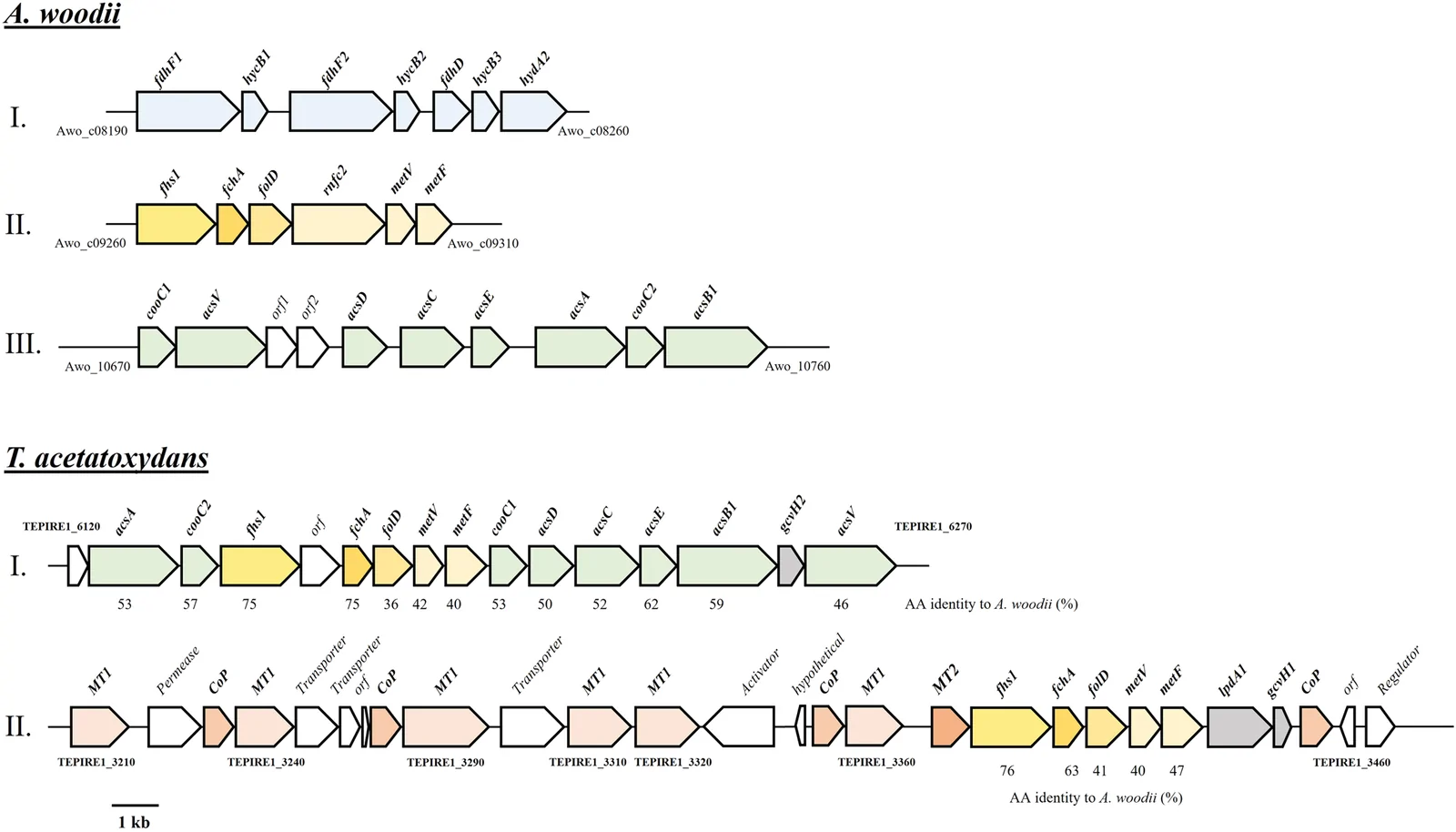
Genetic organization of the Wood–Ljungdahl pathway encoding gene cluster of *T. acetatoxydans* in comparison to *A. woodii*. The genome of *A. woodii* harbors three WLW-encoding gene clusters (I. Hydrogen-dependent CO_2_-reductase encoding genes; II. Methyl branch encoding genes; III. CO dehydrogenase/acetyl-CoA synthase complex encoding genes), while the genome of *T. acetatoxydans* harbors two WLP-encoding gene clusters. Cluster I contains all genes encoding the WLP (CO dehydrogenase, *acsA*; nickel-insertion accessory protein, *cooC1*; formyl-THF synthetase, *fhs1*; methenyl-THF cyclohydrolase, *fchA*; methylene-THF reductase, *metVF*; nickel-insertion accessory protein, *cooC2*; corrinoid-iron sulfur protein, *acsCD*; methyltransferase, *acsE*, acetyl-CoA synthase, *acsB1*, corrinoid activation/regeneration protein, *acsV*) with exception of a formate dehydrogenase-encoding gene. Cluster II harbors genes encoding the methyl branch of the WLP (*fhs1*, *foldD*, *metV*, *metF*) as well as genes encoding multiple methyltransferase systems (MTI, CoP, MTII) and different transporters. Additionally, genes encoding a glycine synthase pathway (*gcvH1*, *gcvH2*, *lpdA1*) are located next to both WLP-encoding gene clusters of *T. acetatoxydans*.

To conserve energy in the form of an electrochemical sodium ion or proton gradient, acetogens commonly use Rnf or Ech complexes.^42^ These complexes use the energy derived from the oxidation of reduced ferredoxin to translocate protons or sodium ions across the membrane. To provide reduced ferredoxin for this reaction, acetogens commonly use the mechanism of electron bifurcation, which couples an exergonic redox reaction with an endergonic redox reaction, such as the reduction of ferredoxin.^43^ The genome of *T. acetatoxydans* harbors genes encoding one Rnf complex as well as one electron-bifurcating hydrogenase complex similar to *A. woodii* (Supporting Information Figure S6). Interestingly, both gene clusters are located next to each other in the genome, only separated by the formate transporter-encoding gene *fdhC*. In addition, *T. acetatoxydans* has a second hydrogenase-encoding gene, located in proximity to the electron-bifurcating hydrogenase-encoding gene cluster (Supporting Information Figure S6). The location of the hydrogenase-encoding gene between two kinase and one phosphatase-encoding genes invites to speculate that *T. acetatoxydans* harbors a sensoric hydrogenase, similar to *Ruminococcus albus*,^44^ which might be used to regulate the expression of the electron-bifurcating hydrogenase gene cluster.

The electrochemical proton or sodium ion gradient is used by an ATP synthase to produce ATP. In sharp contrast to *A. woodii*, the genome of *T. acetatoxydans* does not encode an F_1_F_O_ ATP synthase complex, which is commonly found in bacteria. Müller et al. (2015)^38^ described ATPases in *T. acetatoxydans* erroneously as V-ATP synthases. V-type enzymes do not synthesize ATP, but their function is to pump ions at the expense of ATP hydrolysis.^45^ Our analyses clearly indicate that they are (archaeal) A_1_A_O_-type enzymes, similar to *Eubacterium limosum* (Supporting Information Figure S7A). These enzymes function *in vivo* as ATP synthases. The genetic organization of the first A_1_A_O_-ATP synthase encoding cluster is even identical to *E. limosum*,^46^ the amino acid identities of the corresponding gene products range from 25 to 79%. The organization of the second gene cluster is also highly similar; only the position of *atpC* is different. The gene products of this gene cluster displayed lower similarities (amino acid identity of 21 to 56%) to the A_1_A_O_ ATP synthase subunits of *E. limosum* than to the gene products of the first A_1_A_O_ ATP synthase encoding gene cluster of *T. acetatoxydans*. To identify the coupling ion used by the ATP synthases of *T. acetatoxydans*, we further analyzed the *c* subunits (Supporting Information Figure S7B), which form the membrane-integral motor of all ATP synthases/ATPases. The *c* subunit of F_1_F_O_ ATP synthases consists of a hairpin structure built by two transmembrane helices, connected by a hydrophilic loop. The presence of a conserved glutamate (or aspartate) within the hairpin structure allows the translocation of protons. For the translocation of Na^+^, the presence of two more conserved amino acids (glutamine and threonine or serine) is required.^45^ Due to a duplication event of the transmembrane helices during evolution, V-type ATPases and some A-type ATP synthases harbor *c* subunits with four transmembrane helices (TMH). Both potential ATP synthases of *T. acetatoxydans* harbor 4-TMH *c* subunits, similar to *E. limosum*.^46^ Comparison of the *c* subunits of *T. acetatoxydans* with *c* subunits from different Na^+^- as well as H^+^-dependent ATP synthases revealed that the *c* subunit of the first cluster harbors only one Na^+^-binding site in the second hairpin, similar to the *c* subunit of *E. limosum* (Supporting Information Figure S7B). On the other site, the *c* subunit of the second ATP synthase has no Na^+^-binding site in any hairpin but a proton-binding site (glutamate) in hairpin 2. Therefore, we conclude that the genome of *T. acetatoxydans* encodes one Na^+^-translocating and one H^+^-translocating A_1_A_O_ ATP synthase. The Na^+^-binding site for the Na^+^-translocating Rnf complex of *A. woodii* has been elucidated recently,^47^ which is built by the subunits RnfA and RnfE. RnfA interacts with Na^+^ by peptide backbone interactions, while the Na^+^-binding site in RnfE is built by functional groups of specific amino acids. The conserved amino acids of RnfE can bind Na^+^ as well as H^+^, which makes the identification of the coupling ion by analysis of the amino acid sequence impossible. Nevertheless, the Na^+^/H^+^-binding amino acids are also conserved in RnfE of *T. acetatoxydans* (Supporting Information Figure S8).

### Growth of *T. acetatoxydans* with Glucose Requires External Formate

The WLP is essential for many (but not all) acetogens as an electron sink during hexose fermentation.^6,36,48–51^ In the absence of a formate dehydrogenase, formate has to be added to the culture as an electron acceptor. Growth of *T. acetatoxydans* on glucose alone was indeed very poor; the growth rate was determined to be 0.006 ± 0.004 h^–1^, and the final OD was only 0.26 ± 0.08. However, growth was restored by the addition of formate (Figure 2); the optical density increased nearly 10-fold (2.1 ± 0.1), and the growth rate increased 20-fold (0.12 ± 0.01 h^–1^) after the addition of 20 mM formate. *T. acetatoxydans* was not able to grow with formate as the only carbon and energy source.

**Figure 2:**
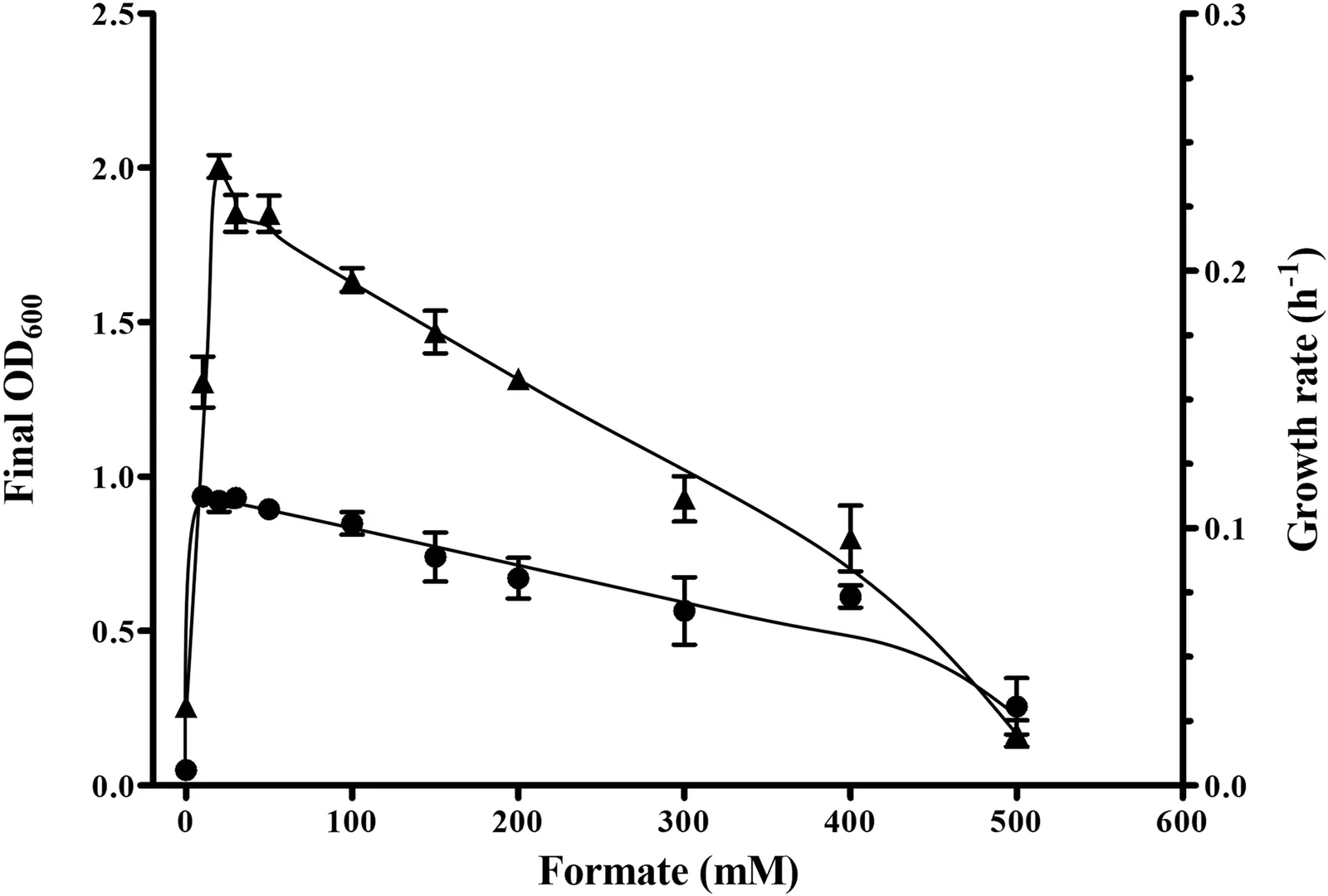
Growth parameters of *T. acetatoxydans* with 20 mM glucose and different formate concentrations as carbon and energy source. *T. acetatoxydans* was grown in 5 mL CO_2_/KHCO_3_-buffered medium with 20 mM glucose and different formate concentrations (0, 10, 20, 30, 50, 100, 150, 200, 300, 400, and 500 mM) as carbon and energy sources at 45 °C. Five % of a preculture of *T. acetatoxydans*, grown with 20 mM glucose and 10 mM formate, was used for inoculation. Final optical densities (▲) and growth rates (●) were determined. All data points are mean ± SEM (Standard Error of the Mean); *N* = 6 independent experiments.

To verify that formate was indeed consumed, metabolite concentrations were determined (Figure 3A). As expected, glucose and formate were simultaneously consumed in stoichiometricamounts during growth (growth rate: 0.13 ± 0.01 h^–1^, final OD_600_: 2.21 ± 0.32), producing 47.14 ± 0.75 mM acetate as the only fermentation product. Furthermore, at the end of fermentation, minor amounts of hydrogen (2.89 ± 1.48 mM) were produced. The fermentation balance was:

1 $$1\;\mathrm{glucose}+1.06\;\mathrm{formate}\to 2.63\;\mathrm{acetate}+0.16\;H_{2}$$

When the concentration of formate was reduced to 3 mM (Figure 3B), the growth rate was similar to growth in the presence of 20 mM formate, but the final optical density was reduced by 67% to 0.72 ± 0.15. Glucose and formate were again consumed simultaneously; however, glucose consumption (3.41 ± 1.23 mM) stopped when formate was completely consumed. Under these conditions, only 15.57 mM acetate was produced. Interestingly, at the end of fermentation, the hydrogen concentration (3.79 ± 0.11 mM) was considerably increased in comparison to formate-rich conditions. The fermentation balance was:

2 $$1\;\mathrm{glucose}+0.72\;\mathrm{formate}\to 2.69\;\mathrm{acetate}+0.95\;H_{2}$$

**Figure 3:**
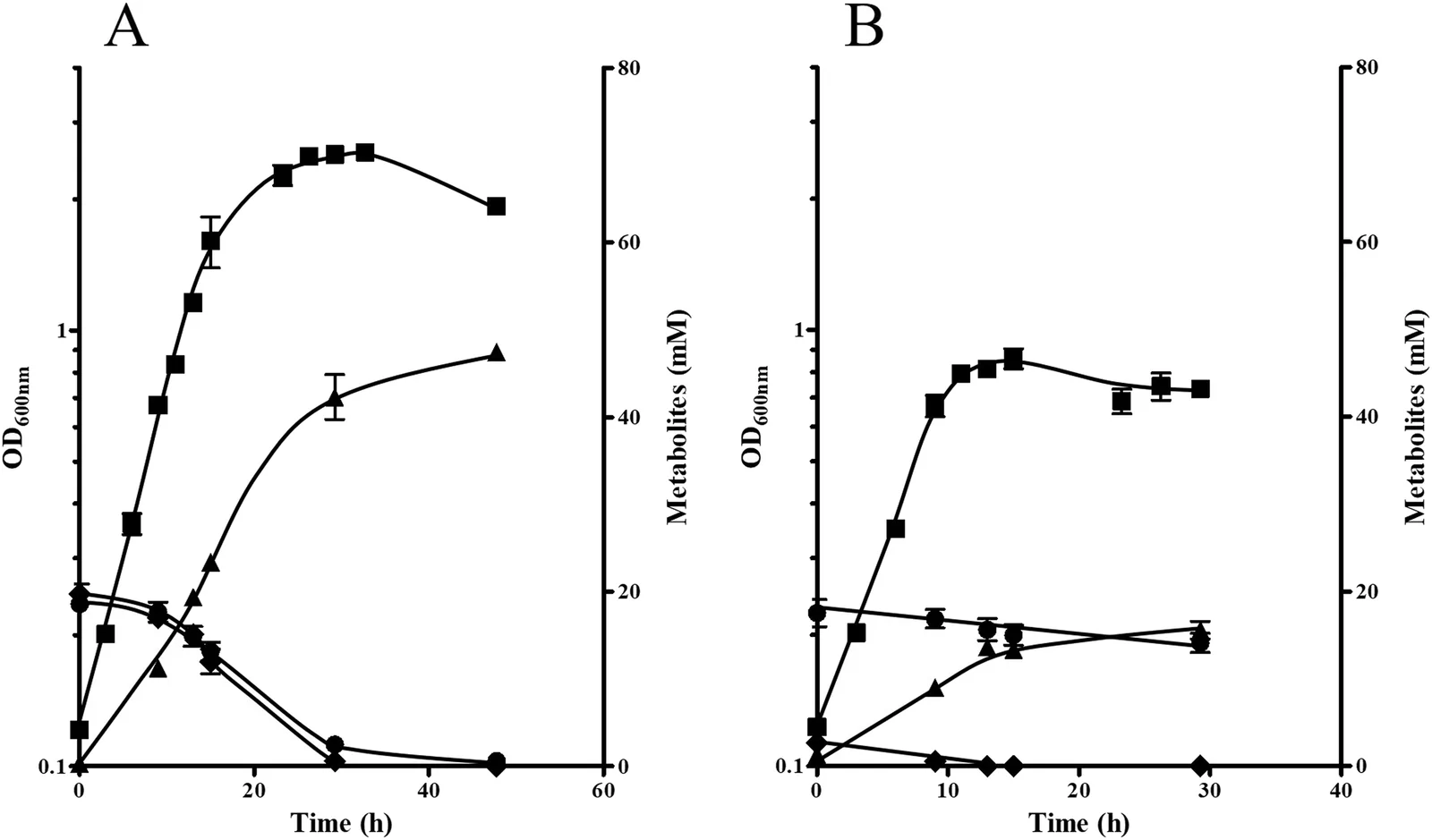
Growth of *T. acetatoxydans* and product formation from glucose in the presence and absence of formate. 5% of a preculture of *T. acetatoxydans* grown with 20 mM glucose and 20 mM formate as substrates was used to inoculate 50 mL of CO_2_/KHCO_3_-buffered medium containing 20 mM glucose and 20 mM formate (A) or 20 mM glucose and 3 mM formate (B) as carbon and energy sources. *T. acetatoxydans* was grown at 45 °C. The optical densities at 600 nm (■) and the concentrations of glucose (●), formate (⧫), and acetate (▲) were determined. All data points mean ± SEM; *N* = 3 independent experiments.

These results demonstrate that *T. acetatoxydans* requires external formate for growth on glucose. In addition, the production of more than two acetates from one glucose is in line with the hypothesis that the formate-dependent WLP of *T. acetatoxydans* is used as an electron sink for glucose oxidation.

The oxidation of glucose and the reoxidation of electron carriers via the WLP are directly coupled to energy conservation (via Rnf/Ech and ATP synthase) in acetogenic bacteria. However, since the genome of *T. acetatoxydans* encodes a Na^+^- as well as a H^+^-dependent ATP synthase, it is unclear whether Na^+^ or H^+^ is used as the coupling ion by the Rnf complex and the ATP synthase. Therefore, we analyzed the effect of Na^+^ on the growth of *T. acetatoxydans*. *T. acetatoxydans* was grown in Na^+^-limited medium (contaminating Na^+^ concentration: 0.84 mM) with glucose and different formate salts (Supporting Information Figure S9). Interestingly, when grown with glucose and potassium formate (0.11 ± 0.01 h^–1^) or ammonium formate (0.10 ± 0.02 h^–1^), growth rates were reduced in comparison to growth in the presence of sodium formate (0.18 ± 0.03 h^–1^); the final optical densities were comparable. When NaCl was added to *T. acetatoxydans* grown on glucose and potassium formate (0.19 ± 0.01 h^–1^) or glucose and ammonium formate (0.21 ± 0.01 h^–1^), growth rates of *T. acetatoxydans* increased and were comparable to growth on glucose and sodium formate. Obviously, Na^+^ stimulated growth of *T. acetatoxydans*.

### Fermentation of Glucose by Resting Cells of *T. acetatoxydans* is Dependent on External Formate

To analyze the metabolism of *T. acetatoxydans* in the absence of biomass production, fermentation experiments with resting cells were performed. In these experiments, cells were washed and resuspended in buffer without ammonia and phosphate to very high densities; these conditions prevent growth but allow catabolic reactions. *T. acetatoxydans* was grown to the late exponential growth phase with glucose and formate as carbon and energy sources, harvested, washed, and resuspended in imidazole buffer. When cells were incubated with glucose as substrate in the absence of formate, 6.01 ± 0.89 mM acetate and 12.0 ± 0.87 mM H_2_ were produced from 3.15 ± 0.09 mM glucose (Figure 4A). The fermentation balance was:

3 $$1\;\mathrm{glucose}\to 1.94\;\mathrm{acetate}+3.81\;H_{2}$$

Again, the WLP was not active under these conditions, and *T. acetatoxydans* seemed to use the production of H_2_ as an electron sink. In the presence of formate, both substrates were simultaneously consumed (Figure 4B), similar to growing cells (see above). The consumption of 3.58 ± 0.20 mM glucose and 4.85 ± 0.88 mM formate led to the production of 11.13 ± 1.92 mM acetate. The amount of acetate increased, and the amount of hydrogen decreased; only 3.03 ± 0.55 mM H_2_ was found, resulting in a fermentation balance of:

4 $$1\;\mathrm{glucose}+1.37\;\mathrm{formate}\to 3.13\;\mathrm{acetate}+0.85\;H_{2}$$

**Figure 4:**
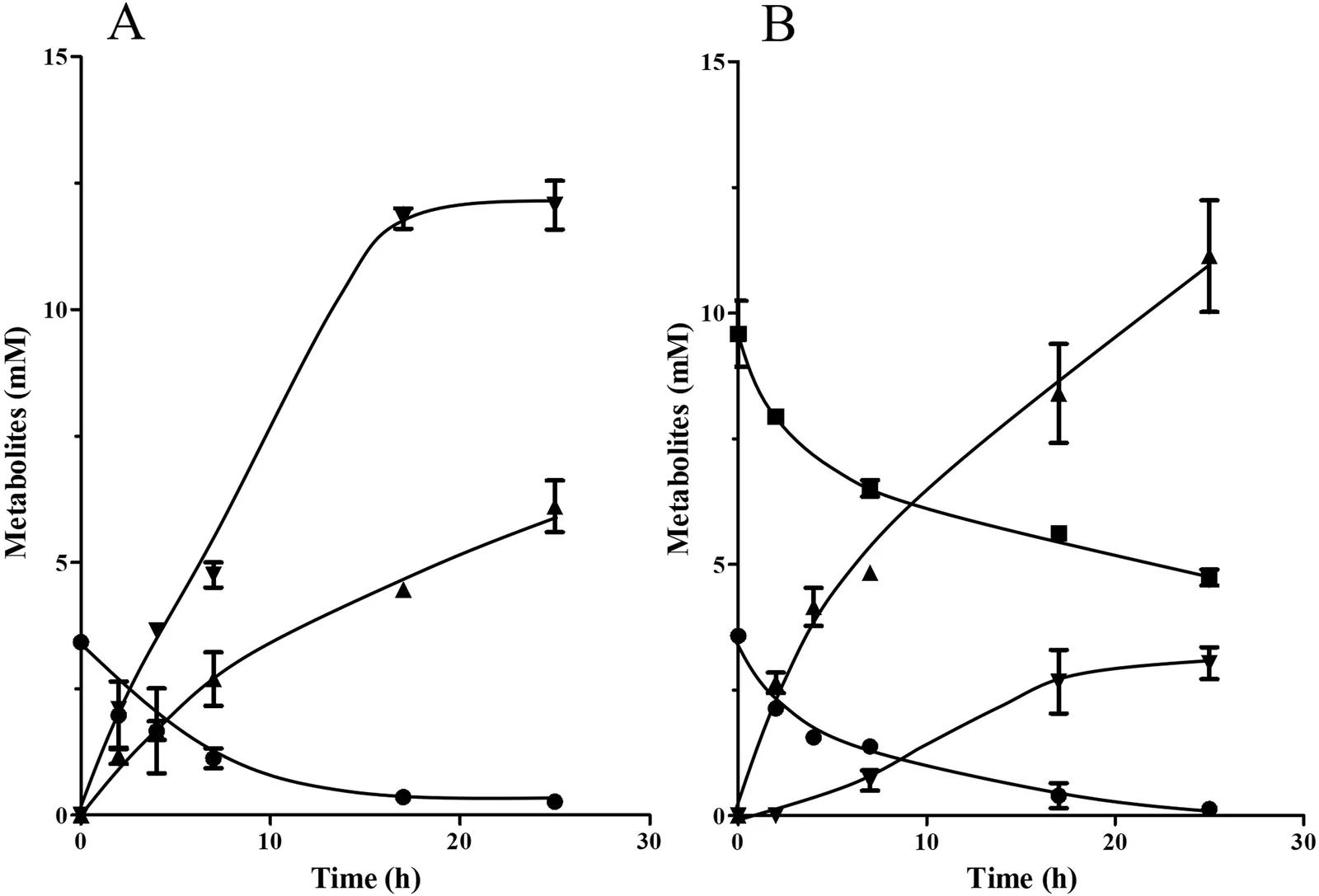
Fermentation of glucose in the presence and absence of formate by resting cells of *T. acetatoxydans*. *T. acetatoxydans* was cultivated in CO_2_/KHCO_3_-bufferend medium with 20 mM glucose and 20 mM formate as carbon and energy sources to the late exponential growth phase. Cells were harvested, washed, and resuspended in 10 mL imidazole buffer to a protein concentration of 1 mg mL^–1^ in 115 mL serum flasks under an N_2_/CO_2_ atmosphere (80/20% [v/v]). Cells were incubated at 45 °C with 3 mM glucose as the only substrate (A) or with 3 mM glucose and 10 mM formate as substrates (B). The concentrations of glucose (●), formate (■), acetate (▲), and H_2_ (▼) were determined at each time point. All data points mean ± SEM; *N* = 3 independent experiments.

The stoichiometry of one glucose consumed and three acetates produced is in line with the fermentation profile of acetogens, which produce a third mole of acetate from glucose via the WLP.

To get further evidence for a functional WLP in *T. acetatoxydans*, we checked whether resting cells were able to form acetate from C1 compounds. As expected, neither H_2_ + CO_2_, formate + CO_2_, nor CO + CO_2_ could be used as substrates for acetate production (Supporting Information Figure S10A–C). However, after the addition of formate, acetate formation from H_2_ + CO_2_ (Supporting Information Figure S10D) or CO + CO_2_ (Supporting Information Figure S10E) was observed. In both cases, the consumption of 1 mol of formate led to the production of 1 mol of acetate, demonstrating once again an active, formate-dependent WLP in *T. acetatoxydans*.

Acetogenesis from formate + H_2_ + CO_2_ by resting cells of *T. acetatoxydans* was also Na^+^-dependent (Figure 5). With no Na^+^ added (contaminating Na^+^-concentration: 184 μM), acetate production from H_2_ + CO_2_ + potassium formate or ammonium formate was negligible but was highly stimulated by NaCl (20 mM) (Figure 5A). The protonophore TCS had no effect on acetate production from H_2_ + CO_2_ + sodium formate or from H_2_ + CO_2_ + potassium formate (Figure 5B), but acetogenesis was nearly completely inhibited by the Na^+^-ionophore ETH2120 (Figure 5B). The experimental data presented clearly are in line with the hypothesis that Na^+^ is used as a coupling ion for energy conservation by *T. acetatoxydans*.

**Figure 5:**
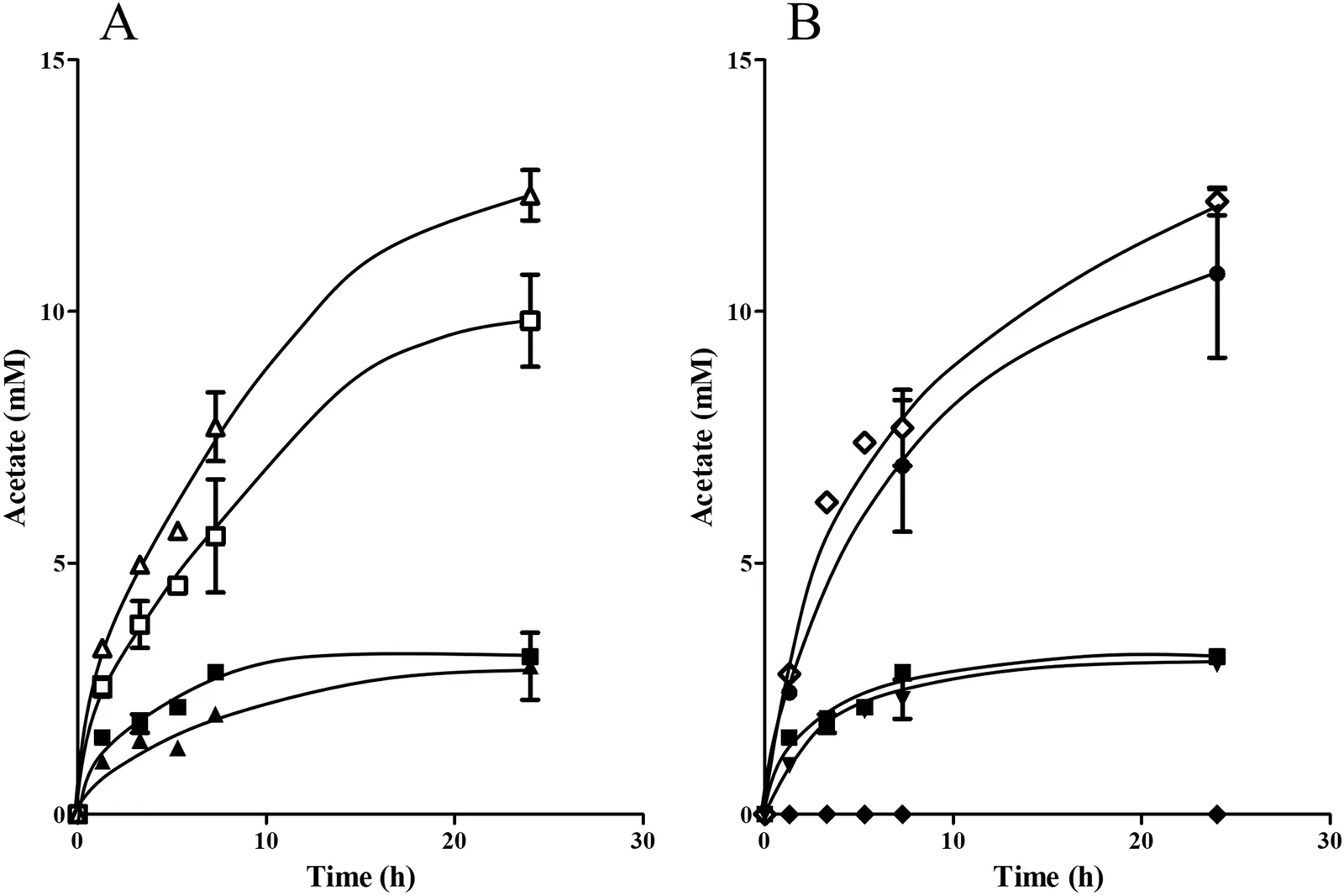
Acetogenesis from formate + H_2_ + CO_2_ by resting cells of *T. acetatoxydans* is Na^+^-dependent. *T. acetatoxydans* was grown in CO_2_/KHCO_3_-buffered medium at 45 °C with 20 mM glucose and 20 mM formate as carbon and energy source (in the presence of 20 mM NaCl) to late exponential growth phase, harvested, washed, and resuspended in 10 mL Na^+^-free imidazole buffer (50 mM imidazole, 60 mM KHCO_3_, 20 mM KCl, 20 mM MgSO_4_, 2 mM DTE, 4.4 μM resazurin, pH 7.0; contaminating Na^+^ concentration: 184 μM) in 115 mL serum flasks to a final protein concentration of 1 mg mL^–1^. Cells were incubated under an H_2_ + CO_2_ atmosphere (1 bar, 100%) at 45 °C with 20 mM potassium formate (■), ammonium formate (▲), with 20 mM potassium formate in the presence of 20 mM NaCl (□) or with 20 mM ammonium formate in the presence of 20 mM NaCl (Δ) (A). Additionally, cells were incubated under a H_2_ + CO_2_ atmosphere (1 bar, 100%) with 20 mM sodium formate (●), potassium formate (■), with 20 mM sodium formate in the presence of 30 μM of the proton-ionophore TCS (◊), with potassium formate and TCS (▼) or with 20 mM sodium formate in the presence of 30 μM of the Na^+^-ionophore ETH2120 (⧫) (B). The concentration of acetate was determined at each point. All data points mean ± SEM; *N* = 2 independent experiments.

### Enzyme Activities in Crude Extract of *T. acetatoxydans*

Next, we determined enzyme activities in crude extract of *T. acetatoxydans* grown on 20 mM glucose and 20 mM formate (Table 1). The methylene-THF-dehydrogenase activity was 2.17 ± 0.12 U mg^–1^ with NADP^+^ as the electron acceptor; NAD^+^ was not reduced with methylene-THF as the electron donor. In addition, we could determine a CO:MV oxidoreductase activity of 5.85 ± 0.41 U mg^–1^ (Table 1). The fact that the only CO dehydrogenase encoded in the genome of *T. acetatoxydans* is part of the CODH/ACS complex of the WLP further confirms the hypothesis of an active WLP during growth. Since the fermentation experiments revealed production as well as consumption of H_2_, it was no surprise that we were also able to determine hydrogenase activity (H_2_:MV oxidoreductase activity of 26.86 ± 2.74 U mg^–1^). As expected, formate dehydrogenase activity could not be measured. Nevertheless, some bacteria can produce formate not only by formate dehydrogenases but by a pyruvate-formate lyase (PFL), which is encoded in the genome of *T. acetatoxydans*.^38^ However, PFL was not active in the crude extract of *T. acetatoxydans*. Instead, we determined a pyruvate-ferredoxin oxidoreductase (PFOR) activity of 1.56 ± 0.10 U mg^–1^ (Table 1). The PFOR is used by most acetogenic bacteria to metabolize pyruvate to acetyl-CoA, since the reaction is coupled to the reduction of ferredoxin, resulting in an energetic advantage (see below).

**Table 1:** Enzyme Activities of Crude Extract of *T. acetatoxydans*

**enzyme activity**	**substrates**	**specific activity (U mg** ^**–1**^ **)** ^a^
hydrogenase	H_2_ + MV	26.86 ± 2.74
CO dehydrogenase	CO + MV	5.85 ± 0.41
MTHF dehydrogenase	methylene-THF + NADP^+^	2.17 ± 0.12
formate dehydrogenase	formate + BV	-
PFOR	pyruvate + CoA + Fd_ox_ + TPP	1.56 ± 0.10
PFL	pyruvate + CoA	-

a Enzyme activities after growth on fructose and formate were determined as described in “Experimental procedures”. MV, Methylviologen; BV, Benzylviologen. All values are mean ± SEM; *N* = 4.

### Utilization of methyl groups by *T. acetatoxydans*

Methylated compounds such as methanol, methylamines, methylsulfides, and methoxylated aromatics are substrates for many acetogens.^18,19^ Since the genome of *T. acetatoxydans* encodes several methyltransferase systems, we asked whether *T. acetatoxydans* can grow on methyl group-containing substrates. All MTI enzymes showed the highest amino acid identities (28 to 44%) to the methyltransferases MtoB1 and MtoB2 of *Methermicoccus shengliensis* (Supporting Information Table S1), which allow this thermophilic methanogenic archaeon to demethylate methoxylated aromatic compounds.^52^ Especially the MT1 enzymes, encoded by TEPIRE1_3210, TEPIRE1_3240 and TEPIRE1_3260, showed high amino acid identities to MtoB1 (42, 42, and 40%) and to MtoB2 (43, 44, and 44%) (Supporting Information Table S1 and Supporting Information Figure S11). These findings suggest that*T. acetatoxydans* grows on methoxylated aromatic compounds. Indeed, we observed growth of *T. acetatoxydans* on syringate (4-hydroxy-3,5-dimethoxybenzoic acid). With 5 mM syringate, cultures grew to a final OD_600_ of 0.18 ± 0.01 (Figure 6). Without syringate, the final OD was only 0.07 ± 0.01. Unexpectedly, during fermentation, formate was not found, and acetate was the only fermentation end product.

**Figure 6:**
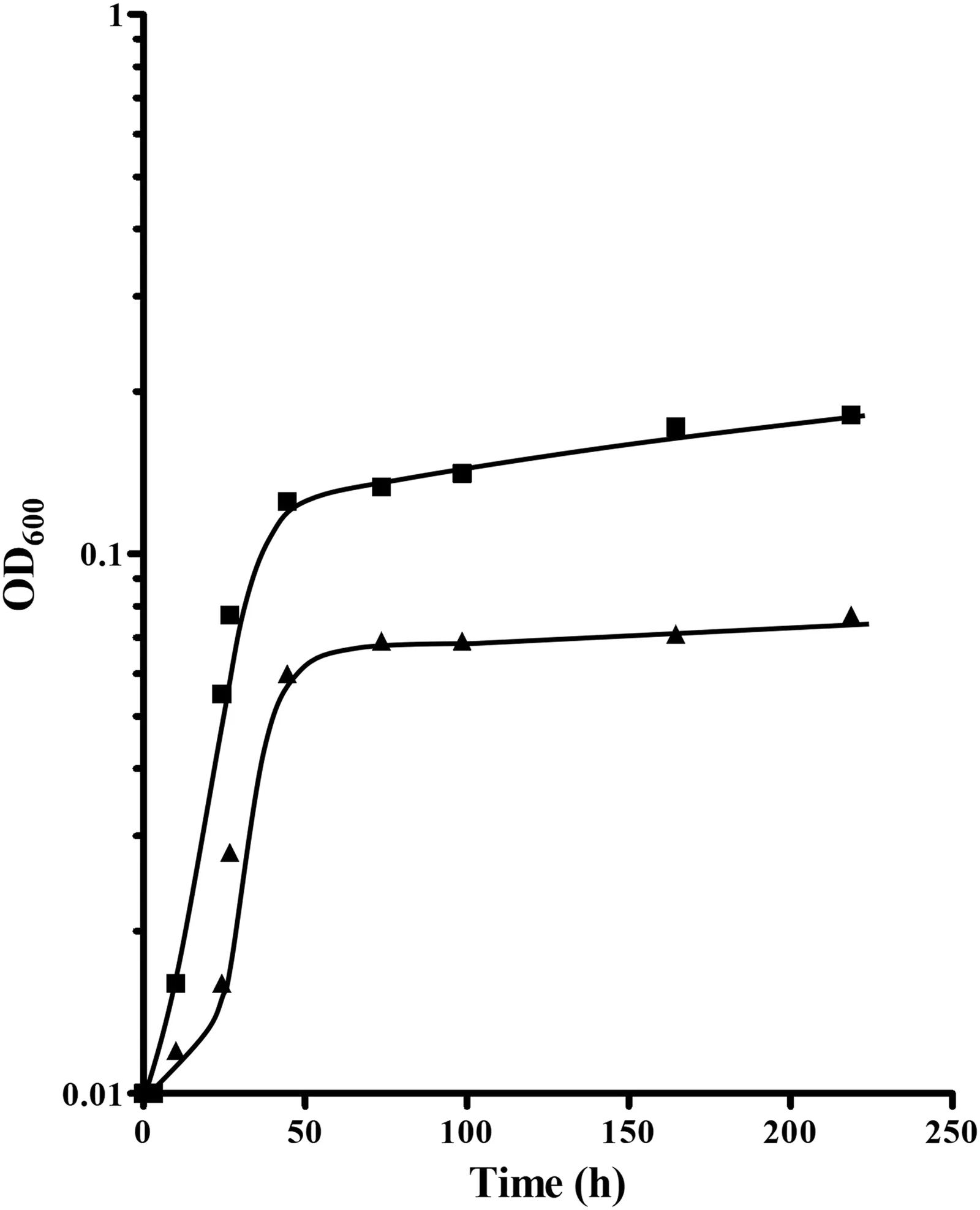
Growth of *T. acetatoxydans* with syringate as carbon and energy source. A preculture of *T. acetatoxydans*, adapted to growth with 5 mM syringate as substrate, was used to inoculate (5%) 5 mL CO_2_/KHCO_3_-buffered medium with 5 mM syringate as carbon and energy source (■) or medium without syringate (▲). The cultures were incubated at 45 °C. One representative growth curve of biological triplicates is shown.

Since formate may have been used as a biosynthetic precursor by the growing cells (theoretically via PFL to acetyl-CoA), we repeated the experiments with resting cells. Therefore, *T. acetatoxydans* was cultivated with 5 mM syringate (+CO_2_) as substrate to the late-exponential growth phase, harvested, washed, and resuspended in imidazole buffer. When incubated with 5 mM syringate, resting cells consumed 1.81 ± 0.48 mM syringate and produced 2.11 ± 0.88 mM acetate and 0.96 ± 0.05 mM formate. H_2_ was not produced (Figure 7A). The fermentation balance was:

5 $$1\;\mathrm{syringate}+1.24\;{\mathrm{CO}}_{2}\to 1.24\;\mathrm{acetate}+0.56\;\mathrm{formate}$$

Interestingly, under an H_2_-atmosphere, resting cells no longer produced formate, but the amount of acetate increased (7.81 ± 1.27 mM) (Figure 7B). The fermentation balance was:

6 $$1\;\mathrm{syringate}+1.83\;{\mathrm{CO}}_{2}\to 1.83\;\mathrm{acetate}+0.00\;\mathrm{formate}$$

In the presence of hydrogen, methyl groups are exclusively condensed with CO, derived from CO_2_ reduction by the CODH/ACS complex, and CoA to acetyl-CoA. In sum, methyl groups are used by *T. acetatoxydans* as a carbon and energy source and are metabolized by the WLP.

**Figure 7:**
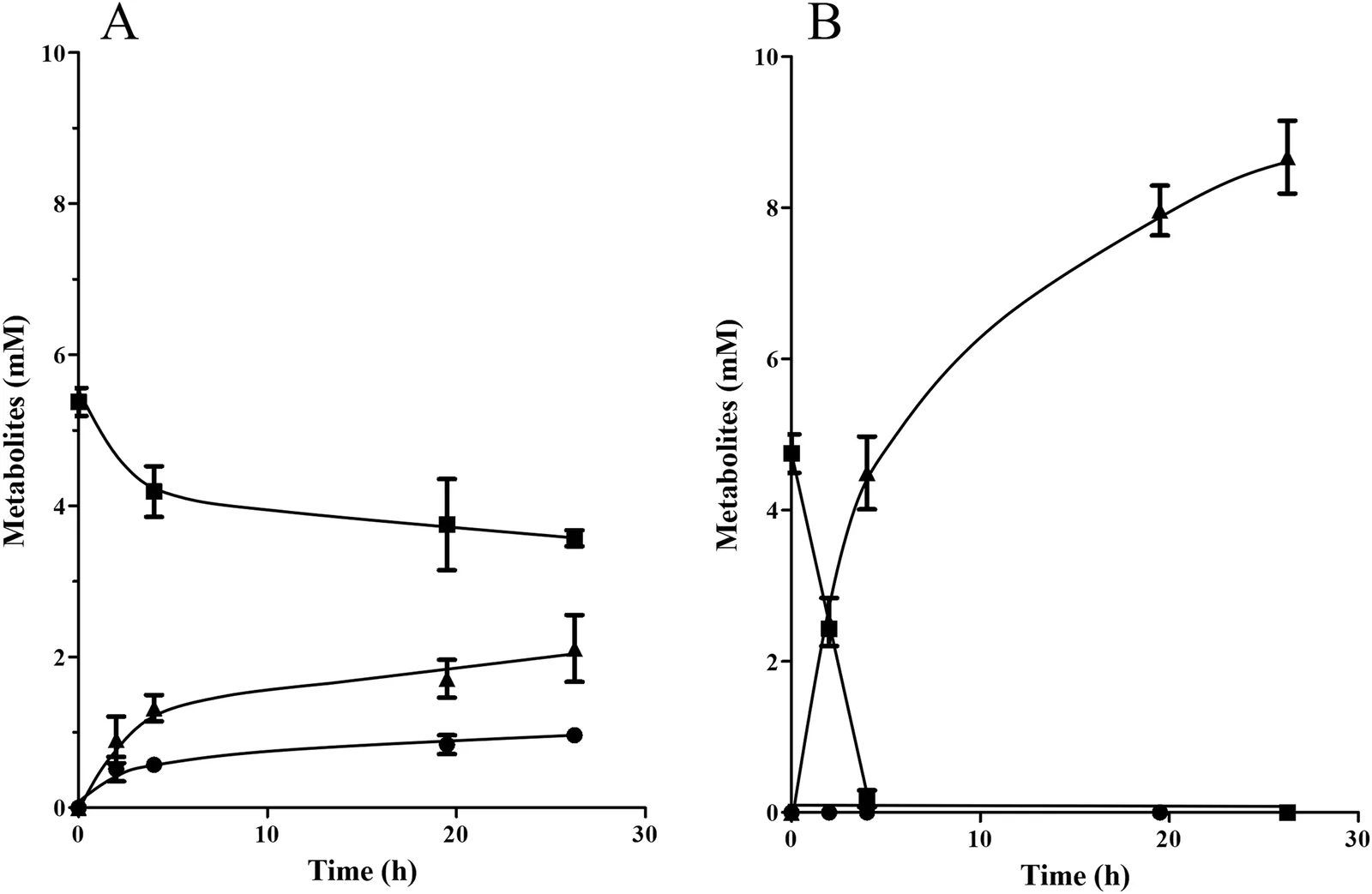
Fermentation of syringate by resting cells of *T. acetatoxydans*. Cells of *T. acetatoxydans*, pre-grown in CO_2_/KHCO_3_-bufferend medium with 5 mM syringate as a carbon and energy source to the late exponential growth phase, were harvested, washed, and resuspended in 10 mL imidazole buffer to a protein concentration of 1 mg mL^–1^ in 115 mL serum flasks. Cells were incubated at 45 °C under a N_2_/CO_2_ atmosphere (80/20% [v/v]) with 5 mM syringate as substrate (A) or with 5 mM syringate and H_2_ + CO_2_ (1 bar, 100%) as substrates (B). The concentrations of acetate (▲), formate (●) and syringate (■) were determined at each time point. All data points mean ± SEM; *N* = 4 independent experiments.

## Discussion

Our data demonstrate that *T. acetatoxydans* has an active WLP devoid of a formate dehydrogenase, which might be a reflection of the presence of formate in a biogas digester. This is in line with recent studies on other acetogens, isolated from formate-rich environments, which have unusual WLPs naturally lacking formate dehydrogenases.^49,53,54^ Since these acetogens cannot catalyze the reduction of CO_2_ to formate and *vice versa*, the methyl branch of the WLP in these bacteria does not start with CO_2_, but formate. This has major effects on the physiology of these acetogens. In some cases, the absence of a formate dehydrogenase results in the requirement for external formate for growth. The formate dehydrogenase-lacking gut acetogen *Clostridium bovifaecis* was not able to grow on sugars as the only carbon and energy sources.^49^ Growth was only possible after addition of formate. The same was observed for an HDCR-deletion (hydrogen-dependent CO_2_-reductase) mutant of the acetogen *Thermoanaerobacter kivui*.^48^ On the other hand, the formate dehydrogenase-lacking acetogen *Blautia luti* can ferment glucose even in the absence of external formate.^36^ This gut acetogen uses a pyruvate-formate lyase to produce formate from pyruvate that is funneled into the WLP as an electron acceptor. Although *T. acetatoxydans* has a PFL gene, the enzyme was not active, and therefore, *T. acetatoxydans* depends on formate cross-feeding for growth on sugars in its natural habitat (Figure 8). Cross-feeding of formate would also allow *T. acetatoxydans* to grow lithotrophically and perform acetogenesis from H_2_ + CO_2_ or CO + CO_2_, as observed for the HDCR mutant of *T. kivui* and for *B. luti*.^48,53^ Hydrogenase as well as CODH activities were present and most likely catalyzed by the electron-bifurcating hydrogenase and the CODH/ACS complex, the only encoded catalytic hydrogenase and CODH, respectively. As in *A. woodii*, oxidation of H_2_ leads to reduction of ferredoxin and reoxidation of ferredoxin by the Na^+^-dependent Rnf complex, followed by the synthesis of ATP. The unusual *c* subunit of the Na^+^-A_1_A_O_ ATP synthase requires less Na^+^ to make an ATP compared to ATP synthases with usual *c* subunits, which allows for ATP synthesis at low thermodynamic driving forces.^55^ The thermodynamic driving force is even lower at alkaline pH values; however, a sodium ion cycle would help to survive at high pH. The second H^+^-A_1_A_O_ ATP synthase might be involved in the regulation of pH homeostasis, since acetogenesis commonly leads to an acidification of the exterior.

**Figure 8:**
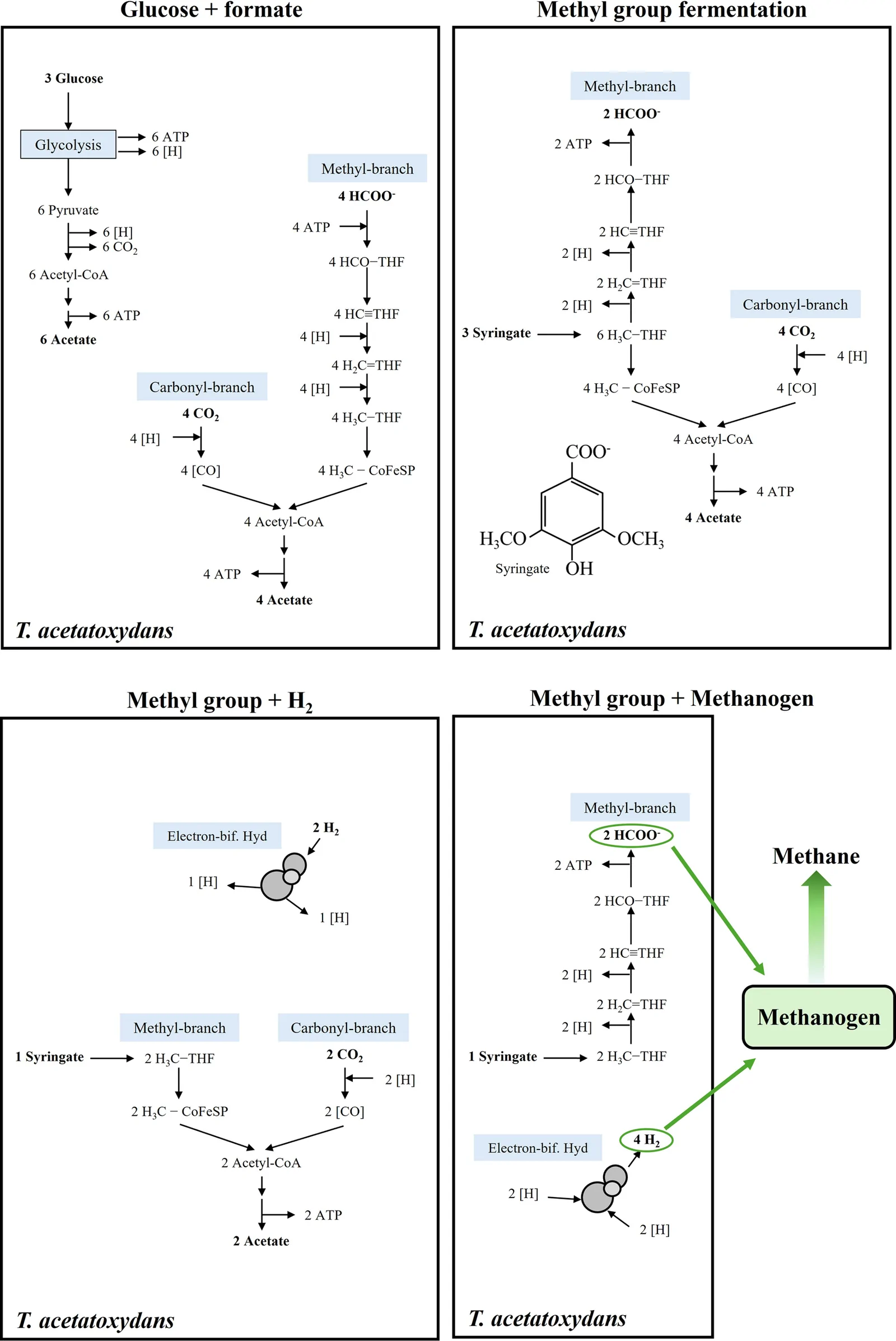
Metabolic schemes for acetogenesis from glucose and formate in *T. acetatoxydans* and the fate of methyl groups under different environmental conditions. Acetogenesis from glucose + formate, from methyl groups from syringate or syringate + H_2_, and nonacetogenic methyl group oxidation in the presence of a methanogen are depicted. For further explanations, see the text. [H], redox equivalent (2e^–^ + 2H^+^). Please note that these are strongly simplified models, not taking into account the nature of the electron carriers and the enzymes such as Rnf or transhydrogenases to balance the different redox carriers required for the metabolic routes. Moreover, some H_2_ is produced from glucose + formate via the electron-bifurcating hydrogenase that is not depicted in the figure.

Oxidation of sugars might not be the only role of *T. acetatoxydans* in the food web of a biogas digester. As a syntrophic acetate-oxidizing bacterium, an important role might also be the supply of CO_2_ and H_2_ for methanogenesis by the oxidation of acetate. However, since *T. acetatoxydans* lacks a formate dehydrogenase, the oxidation of one mole of acetate results in the production of only one mol of CO_2_ and three mol of H_2_, but also one mol of formate. In general, formate and H_2_ are common electron carriers in different ecosystems with similar characteristics,^56^ since both have almost identical redox potentials (E^0^’[H_2_/2H^+^] = −414 mV, E^0^′[formate/CO_2_] = −432 mV) and are stoichiometrically equivalent.^57^ However, formate has a better solubility than H_2_ and can diffuse over long distances through an aqueous phase.^58^ Therefore, it is no surprise that many methanogens can effectively utilize H_2_ and formate as electron donors for methanogenesis.

Another novel finding of this presentation is that *T. acetatoxydans* can grow on syringate, which is present in plant material and readily available as substrates in a biogas digester.^59^ However, it does not cleave the aromatic ring structure but only uses the methyl groups that are channeled into the WLP. Therefore, a prominent role of *T. acetatoxydans* in the biogas digester may be acetogenesis, formatogenesis, and hydrogenogenesis from methoxylated plant material. Demethylation of methoxylated aromatics certainly is an overlooked activity in biogas plants, but acetogens are well-known for this ability. In the case of *T. acetatoxydans*, methyl groups are, due to the lack of a formate dehydrogenase, oxidized to formate, yielding four electrons (Figure 8); the four electrons are used to reduce two mol of CO_2_ to CO, which is condensed with two more methyl groups to acetate (Figure 8). The determined stoichiometry of acetogenesis from syringate (eq 5) suggests that both methyl groups of syringate are used for acetogenesis, yielding gallate (3,4,5-trihydroxybenzoate) as the product. This was also observed in *Desulfitobacterium* species.^60,61^ The demethylation of syringate could be catalyzed by one or two MTI enzymes. This would leave 4 or 3 MTI enzymes whose substrate still needs to be identified. Methanol can be excluded since *T. acetatoxydans* does not grow on methanol, and the low similarities (19–21%) of the enzymes to MTI from methanol (Awo_c22760) suggest other methoxylated substrates for *T. acetatoxydans*.

We have to conclude that the use of methoxylated aromatic compounds makes *T. acetatoxydans* an important player in the biodigester food web. In the presence of H_2_ as an external electron donor, *T. acetatoxydans* exclusively reduced the methyl groups to acetate in a “reduction only” mode (Figure 8), as described for *A. woodii*.^19^ However, in a biogas digester, the fate of methyl groups might be different. Due to the presence of methanogens, methyl groups might be completely oxidized to formate and H_2_ in a “syntrophic oxidation only” mode (Figure 8). Such a fermentation mode has already been described for *A. woodii*, which completely oxidized methyl groups to CO_2_ in the presence of a methanogen^62^ or in the presence of caffeate as an alternative electron acceptor^19^ and additionally showed nonacetogenic growth on methyl group-containing phenyl acrylates.^17^ Therefore, the major role of *T. acetatoxydans* in a biogas digester might be syntrophic acetate oxidation as well as syntrophic methyl group oxidation, to produce formate and H_2_ as electron donors used for methanogenesis.

## Supplementary Material



## Data Availability

All data from this study are available within the article and its Supporting Information. The raw data are available from the authors upon reasonable request.

## Notes

Supporting Information Figure S1. Comparison of the Fhs amino acid sequences of *T. acetatoxydans* and *A. woodii*. Supporting Information Figure S2. Comparison of the FchA amino acid sequences of *T. acetatoxydans* and *A. woodii*. Supporting Information Figure S3. Comparison of the FolD amino acid sequences of *T. acetatoxydans* and *A. woodii*. Supporting Information Figure S4. Comparison of the MetV amino acid sequences of *T. acetatoxydans* and *A. woodii*. Supporting Information Figure S5. Comparison of the MetF amino acid sequences of *T. acetatoxydans* and *A. woodii*. Supporting Information Figure S6. Genetic organization of the Rnf complex and electron-bifurcating hydrogenase-encoding genes of *T. acetatoxydans* in comparison to *A. woodii*. Supporting Information Figure S7. Comparison of the genetic organization of the A_1_A_O_ ATP synthase encoding gene cluster of *T. acetatoxydans* and *E. limosum* and comparison of the ATP synthase c subunits. Supporting Information Figure S8. Comparison of the RnfE subunits of *T. acetatoxydans* with RnfE subunits of other Rnf-complexes. Supporting Information Figure S9. Growth of *T. acetatoxydans* with glucose and formate as carbon and energy sources is stimulated by Na^+^. Supporting Information Figure S10. Acetogenesis from C1 compounds by resting cells of *T. acetatoxydans*. Supporting Information Figure S11. Comparison of the amino acid sequences of the MTI methyltransferases of *T. acetatoxydans* to the methyltransferases MotB1 and MotB2 of *M. shengliensis*. Supporting Information Table S1. Amino acid identities of MT1 enzymes of *T. acetatoxydans* in comparison to the methyltransferases MtoB1 and MtoB2 of *M. shengliensis* (PDF)

## References

[1] Schink B. (2002). Synergistic
interactions in the microbial world. Antonie
van Leeuwenhoek.

[2] Morris B. E., Henneberger R., Huber H., Moissl-Eichinger C. (2013). Microbial
syntrophy: interaction for the common good. FEMS Microbiol. Rev..

[3] Ferry J. G. (1992). Biochemistry
of methanogenesis. Crit. Rev. Biochem. Mol.
Biol..

[4] Bueno
de Mesquita C. P., Wu D., Tringe S. G. (2023). Methyl-based methanogenesis:
an ecological and genomic review. Microbiol.
Mol. Biol. Rev..

[5] Schuchmann K., Müller V. (2016). Energetics and application of heterotrophy in acetogenic
bacteria. Appl. Environ. Microbiol..

[6] Müller V. (2003). Energy conservation
in acetogenic bacteria. Appl. Environ. Microbiol..

[7] Schaupp A., Ljungdahl L. G. (1974). Purification
and properties of acetate kinase from *Clostridium thermoaceticum*. Arch.
Microbiol..

[8] Müller, V. ; Imkamp, F. ; Rauwolf, A. ; Küsel, K. ; Drake, H. L. Molecular and cellular biology of acetogenic bacteria. In Strict and Facultative Anaerobes Medical and Environmental Aspects; Nakano, M. M. ; Zuber, P. , Eds.; Horizon Biosciences: Norfolk, 2004; pp 251–281.

[9] Drake H. L., Gößner A. S., Daniel S. L. (2008). Old acetogens, new
light. Ann. N.Y. Acad. Sci..

[10] Ragsdale S. W. (2008). Enzymology
of the Wood-Ljungdahl pathway of acetogenesis. Ann. N.Y. Acad. Sci..

[11] Himes R. H., Harmony J. A., Rabinowitz J. C. (1973). Formyltetrahydrofolate synthetas. CRC Crit. Rev. Biochem..

[12] Ragsdale S. W., Ljungdahl L. G. (1984). Purification
and properties of NAD-dependent 5,10-methylenetetrahydrofolate
dehydrogenase from *Acetobacterium woodii*. J. Biol. Chem..

[13] Lovell C. R., Przybyla A., Ljungdahl L. G. (1988). Cloning
and expression in *Escherichia coli* of
the *Clostridium thermoaceticum* gene encoding thermostable
formyltetrahydrofolate synthetase. Arch. Microbiol..

[14] Pezacka E., Wood H. G. (1984). The synthesis of
acetyl-CoA by *Clostridium
thermoaceticum* from carbon dioxide, hydrogen, coenzyme A
and methyltetrahydrofolate. Arch. Microbiol..

[15] Raybuck S. A., Bastian N. R., Orme-Johnson W. H., Walsh C. T. (1988). Kinetic characterization
of the carbon monoxide-acetyl-CoA (carbonyl group) exchange activity
of the acetyl-CoA synthesizing CO dehydrogenase from *Clostridium
thermoaceticum*. Biochemistry.

[16] Seravalli J., Kumar M., Lu W. P., Ragsdale S. W. (1997). Mechanism of carbon
monoxide oxidation by the carbon monoxide dehydrogenase/acetyl-CoA
synthase from *Clostridium thermoaceticum*: Kinetic
characterization of the intermediates. Biochemistry.

[17] Bache R., Pfennig N. (1981). Selective isolation
of *Acetobacterium
woodii* on methoxylated aromatic acids and determination
of growth yields. Arch. Microbiol..

[18] Kremp F., Müller V. (2021). Methanol and
methyl group conversion in acetogenic
bacteria: biochemistry, physiology and application. FEMS Microbiol. Rev..

[19] Litty D., Kremp F., Müller V. (2022). One substrate,
many fates: different
ways of methanol utilization in the acetogen *Acetobacterium
woodii*. Environ. Microbiol..

[20] Hattori S. (2008). Syntrophic
acetate-oxidizing microbes in methanogenic environments. Microbes Environ..

[21] Koster I. W., Lettinga G. (1984). The influence of ammonium-nitrogen on the specific
activity of pelletized methanogenic sludge. Agric Wastes.

[22] Sprott G. D., Patel G. B. (1986). Ammonia toxicity in pure cultures of methanogenic bacteria. Syst. Appl. Microbiol..

[23] Hunik J. H., Hamelers H. V. M., Koster I. W. (1990). Growth-rate inhibition
of acetoclastic
methanogens by ammonia and pH in poultry manure digestion. Biol. Wastes.

[24] Hajarnis S. R., Ranade D. R. (1993). Revival of ammonia inhibited cultures
of *Methanobacterium
bryantii* and *Methanosarcina barkeri*. J. Ferment. Bioeng..

[25] Westerholm M., Roos S., Schnürer A. (2011). *Tepidanaerobacter
acetatoxydans* sp. nov., an anaerobic, syntrophic acetate-oxidizing
bacterium isolated from two ammonium-enriched mesophilic methanogenic
processes. Syst. Appl. Microbiol..

[26] Heise R., Müller V., Gottschalk G. (1989). Sodium dependence of acetate formation
by the acetogenic bacterium *Acetobacterium woodii*. J. Bacteriol..

[27] Moon J., Müller V. (2021). Physiology
and genetics of ethanologenesis in the acetogenic
bacterium *Acetobacterium woodii*. Environ. Microbiol..

[28] Schmidt K., Liaaen-Jensen S., Schlegel H. G. (1963). Die Carotinoide der *Thiorhodaceae*. Arch. Mikrobiol..

[29] Trifunović D., Moon J., Poehlein A., Daniel R., Müller V. (2021). Growth of
the acetogenic bacterium *Acetobacterium woodii* on glycerol and dihydroxyacetone. Environ.
Microbiol..

[30] Weghoff M. C., Müller V. (2016). CO metabolism in the thermophilic acetogen *Thermoanaerobacter kivui*. Appl.
Environ. Microbiol..

[31] Schwarz F. M., Ciurus S., Jain S., Baum C., Wiechmann A., Basen M., Müller V. (2020). Revealing formate production from
carbon monoxide in wild type and mutants of Rnf- and Ech-containing
acetogens, *Acetobacterium woodii* and *Thermoanaerobacter kivui*. Microb.
Biotechnol..

[32] Wiechmann A., Müller V. (2021). Energy conservation in the acetogenic bacterium *Clostridium aceticum*. Microorganisms.

[33] Bradford M. M. (1976). A rapid
and sensitive method for the quantification of microgram quantities
of protein utilizing the principle of proteine-dye-binding. Anal. Biochem..

[34] Wang S., Huang H., Kahnt J., Müller A. P., Köpke M., Thauer R. K. (2013). NADP-specific electron-bifurcating
[FeFe]-hydrogenase in a functional complex with formate dehydrogenase
in *Clostridium autoethanogenum* grown on CO. J. Bacteriol..

[35] Schwarz F. M., Schuchmann K., Müller V. (2018). Hydrogenation of CO_2_ at
ambient pressure catalyzed by a highly active thermostable biocatalyst. Biotechnol. Biofuels.

[36] Trischler R., Müller V. (2026). Formate as
electron carrier in the gut acetogen *Blautia luti*: a model for electron transfer in the
gut microbiome. Gut Microbes.

[37] Schönheit P., Wäscher C., Thauer R. K. (1978). A rapid procedure
for the purification
of ferredoxin from Clostridia using polyethylenimine. FEBS Lett..

[38] Müller B., Manzoor S., Niazi A., Bongcam-Rudloff E., Schnürer A. (2015). Genome-guided analysis of physiological capacities
of *Tepidanaerobacter acetatoxydans* provides
insights into environmental adaptations and syntrophic acetate oxidation. PLoS One.

[39] Öppinger C., Kremp F., Müller V. (2022). Is reduced
ferredoxin the physiological
electron donor for MetVF-type methylenetetrahydrofolate reductases
in acetogenesis? A hypothesis. Int. Microbiol..

[40] Song Y., Lee J. S., Shin J., Lee G. M., Jin S., Kang S. (2020). Functional
cooperation of the glycine synthase-reductase
and Wood-Ljungdahl pathways for autotrophic growth of *Clostridium
drakei*. Proc. Natl. Acad. Sci. U. S.
A..

[41] Moon J., Dönig J., Kramer S., Poehlein A., Daniel R., Müller V. (2021). Formate metabolism in the acetogenic bacterium *Acetobacterium woodii*. Environ.
Microbiol..

[42] Schuchmann K., Müller V. (2014). Autotrophy at the thermodynamic limit of life: a model
for energy conservation in acetogenic bacteria. Nat. Rev. Microbiol..

[43] Müller V., Chowdhury N. P., Basen M. (2018). Electron bifurcation: a long-hidden
energy-coupling mechanism. Annu. Rev. Microbiol..

[44] Zheng Y., Kahnt J., Kwon I. H., Mackie R. I., Thauer R. K. (2014). Hydrogen
formation and its regulation in *Ruminococcus albus*: involvement of an electron-bifurcating [FeFe]-hydrogenase, of a
non-electron-bifurcating [FeFe]-hydrogenase, and of a putative hydrogen-sensing
[FeFe]-hydrogenase. J. Bacteriol..

[45] Müller V., Grüber G. (2003). ATP synthases:
structure, function and evolution of
unique energy converters. Cell. Mol. Life Sci..

[46] Litty D., Müller V. (2020). A Na^+^ A_1_ A_O_ ATP synthase
with a V-type *c* subunit in a mesophilic bacterium. FEBS J..

[47] Kumar A., Roth J., Kim H., Saura P., Bohn S., Reif-Trauttmansdorff T. (2025). Molecular principles of redox-coupled
sodium pumping of the ancient Rnf machinery. Nat. Commun..

[48] Jain S., Dietrich H. M., Müller V., Basen M. (2020). Formate is required
for growth of the thermophilic acetogenic bacterium *Thermoanaerobacter kivui* lacking hydrogen-dependent
carbon dioxide reductase (HDCR). Front. Microbiol..

[49] Yao Y., Fu B., Han D., Zhang Y., Liu H. (2020). Formate-dependent
acetogenic
utilization of glucose by the fecal acetogen *Clostridium
bovifaecis*. Appl. Environ. Microbiol..

[50] Moon J., Waschinger L. M., Müller V. (2023). Lactate formation
from fructose or
C1 compounds in the acetogen *Acetobacterium woodii* by metabolic engineering. Appl. Microbiol.
Biotechnol..

[51] Trischler R., Poehlein A., Daniel R., Müller V. (2023). Ethanologenesis
from glycerol by the gut acetogen *Blautia schinkii*. Environ. Microbiol..

[52] Kurth J. M., Nobu M. K., Tamaki H., de Jonge N., Berger S., Jetten M. S. M. (2021). Methanogenic archaea use a bacteria-like methyltransferase
system to demethoxylate aromatic compounds. ISME J..

[53] Trischler R., Roth J., Sorbara M. T., Schlegel X., Müller V. (2022). A functional
Wood-Ljungdahl pathway devoid of a formate dehydrogenase in the gut
acetogens *Blautia wexlerae*, *Blautia
luti* and beyond. Environ. Microbiol..

[54] Li Q., Huo J., Ni G., Zhang F., Zhang S., Zhang X. (2025). Reductive
acetogenesis is a dominant process in the ruminant hindgut. Microbiome.

[55] Litty D., Müller V. (2022). ATP synthesis
in an ancient ATP synthase at low driving
forces. Proc. Natl. Acad. Sci. U. S. A..

[56] Schink B., Montag D., Keller A., Müller N. (2017). Hydrogen or
formate: Alternative key players in methanogenic degradation. Environ. Microbiol. Rep..

[57] Thauer R. K., Jungermann K., Decker K. (1977). Energy conservation in chemotrophic
anaerobic bacteria. Bact. Rev..

[58] Schink B. (1997). Energetics
of syntrophic cooperation in methanogenic degradation. Microbiol. Mol. Biol. Rev..

[59] Phelps C. D., Young L. Y. (1997). Microbial metabolism
of the plant phenolic compounds
ferulic and syringic acids under three anaerobic conditions. Microb. Ecol..

[60] Studenik S., Vogel M., Diekert G. (2012). Characterization of an O-demethylase
of *Desulfitobacterium hafniense* DCB-2. J. Bacteriol..

[61] Mingo F. S., Studenik S., Diekert G. (2014). Conversion of phenyl methyl ethers
by *Desulfitobacterium* spp. and screening for the
genes involved. FEMS Microbiol. Ecol..

[62] Winter J. U., Wolfe R. S. (1980). Methane formation
from fructose by syntrophic associations
of *Acetobacterium woodii* and different
strains of methanogens. Arch. Microbiol..

