# Supplementary material for: Formate- and Sodium Ion-Dependent Wood–Ljungdahl Pathway Involved in Organo‑, Litho‑, and Methylotrophic Growth of Tepidanaerobacter acetatoxydans
Source: Environ Sci Technol. 2026 Aug 25;60(35):24792–803. doi: 10.1021/acs.est.6c09717 (PMC13564433; doi:10.1021/acs.est.6c09717)
Supplement: Supplementary file 1 [file es6c09717_si_001.pdf]

Supplementary Data

A formate- and sodium ion-dependent Wood-Ljungdahl pathway involved in organo-, litho- and methylotrophic growth of *Tepidanaerobacter acetatoxydans*

Raphael Trischler[1], Lennox J.-V. Fletcher[1], Volker Müller[1#]

[1] *Molecular Microbiology & Bioenergetics, Institute of Molecular Biosciences, Johann Wolfgang Goethe University, Max-von-Laue Str. 9, D-60438 Frankfurt, Germany*

#Corresponding author. Mailing address: Department of Molecular Microbiology & Bioenergetics, Institute of Molecular Biosciences, Johann Wolfgang Goethe University, Max-von-Laue-Str. 9, D-60438 Frankfurt, Germany. Phone: 49-69-79829507. Fax: 49-69-79829306. E-mail: vmueller@bio.uni-frankfurt.de.

```
A. woodii             MGFKSDIEIAQEATPQDIREIAKKLGLTEDDLDLYGKYKAKVDYNLLKKSTG-KKARLIL    59
T. acetatoxydans 1    MSYKSDIEIAQEAKIEHIKDVATKIGLCEDDIEYYGKYKAKIDYNLLKRFEDKKDAKLIL    60
T. acetatoxydans 2    MTFKSDIEIAQSVKLQDIREIAAKLGLTEDDIDLYGKYKAKVDYNLLNNCNG-KKAKLIL    59
                      * :********... :.*::* *:** ***:: *******:*****:.  . *.*:***

A. woodii             TTAINPTPAGEGKTTTTIGVADGLSRIGKNTLVALREPSLGPVFGVKGGAAGGGYAQVVP    119
T. acetatoxydans 1    TTAINPTPAGEGKTTTTVGLGDALRRLGKNAMIALREPSLGPVFGIKGGAAGGGYAQVVP    120
T. acetatoxydans 2    TTAITPTPAGEGKTTTTIGAADALTRLGKKTIVALREPSLGPVFGIKGGAAGGGYAQVVP    119
                      ****.************:* .*.* *:**:::************:**************

A. woodii             MEDINLHFTGDFHAIGAANNLLAAMLDNHIKQGNELKIDAKKITWRRCVDMNDRQLRNIV    179
T. acetatoxydans 1    MEDINLHFTGDFHAIGAANNLLAAMIDNHIYQGNELNIDPRRITWKRCVDMNDRQLRFVV    180
T. acetatoxydans 2    MEDINLHFTGDIHAITAANNLLAAMVDNHIFQGNQLNIDTRRVVWRRAVDINDRQLRFVI    179
                      ***********:*** *********:**** ***:*:** :::.*:*.**:****** ::

A. woodii             DGLGGSGDGVVREDGFDITVASEVMAAFCLSSDISDLKERLGRIIVAYSFTGEPVTAEQL    239
T. acetatoxydans 1    DGLGGKANGTPREDGYDITVASEIMAVFCLANDMEDLKNRLARIIIGYTYDGKPVTAGQL    240
T. acetatoxydans 2    DGLGGKANGVPREDGFDITVASEVMAIFCLANDIMDLKERLAKIVVAYDREGKPVTAGDL    239
                      *****..:*. ****:*******:** ***:.*: ***:**.:*::.*   *:**** :*

A. woodii             KANGAMAALLKDALKPNLVQTLEGTPAFIHGGPFANIAHGCNSVIATRMAMHFADYVVTE    299
T. acetatoxydans 1    KAQGAMAALLKDAFKPNLVQTLEGTPAFVHGGPFANIAHGCNSIIATKMALKLADYVVTE    300
T. acetatoxydans 2    KAQGAMAALLKDALKPNLVQTLEGTPAFVHGGPFANIAHGCNSVIATKMAMHLADYVVTE    299
                      **:**********:**************:**************:***:**:::*******

A. woodii             GGFGADLGAEKFLDIKCRMANLKPDAVIIVATVRALKYNGGVAKADLNNENLEALKAGLP    359
T. acetatoxydans 1    AGFGADLGAEKFLDIKCRMADIRPDAVVIVATIRALKYNGGVKKEDLNQENLDALKKGLP    360
T. acetatoxydans 2    AGFGADLGAEKFIDIKCRLAGLKPDAVIIVATVRALKYNGGLAKADLEKEDLNALAKGIP    359
                      .***********:*****:*.::****:****:********: * **::*:*:**  *:*

A. woodii             NLLKHVENITQVFKLPAVVAINEFPLDTEAELQLVKSECQKLGVNVAISQVWAKGGEGGE    419
T. acetatoxydans 1    NLLKHVENITEKYGIPAVVAINQFPTDTERELALVQEECNRLGVNAVLSEVWAKGGEGGL    420
T. acetatoxydans 2    NLLKHVENITQVFKLPAVVAINRFPQDTEAELKLVEDRCNELGVNVALSEVWAKGGEGGI    419
                      **********: : :*******.** *** ** **:..*:.****..:*:*********

A. woodii             ELAKEVVRLIDES---EGNFEYCYDLDIPIKEKIETIATRIYGADGVDFTPAAAKEMDRL    476
T. acetatoxydans 1    ELAKEVVRIIEEG---KNNFKPIYDLDMGIADKITTIAKEIYGADGVEFAPAALKEINTL    477
T. acetatoxydans 2    ALAEELIRLTEDNKASNKGLAYVYELDMPIKKKIRAISQKIYGADDVMFTDKALKEIANL    479
                       **:*::*: ::.   : .:   *:**: * .** :*: .*****.* *:  * **:  *

A. woodii             TALGFDKVPICMAKTQYSLTDDATKLGRPTGFKITVRQLTISAGAGFIIALTGEIMKMPG    536
T. acetatoxydans 1    EELGFKNVPVCIAKTQYSLTDDPKLLGRPTGFKINVRNVKISAGAGFVVALTGAIMTMPG    537
T. acetatoxydans 2    EKLGFGKMPVCIAKTQYSLTDDPKKLGRPSGFNITVRDVSVSAGAGFIVAVTGDIMKMPG    539
                        *** ::*:*:********** . ****:**:*.**::.:******::*:** **.***

A. woodii             LPKVPAAEKIDVDENGVIAGLF    558
T. acetatoxydans 1    LPKRPAAEKIDVDVNGKIAGLF    559
T. acetatoxydans 2    LPKVPAAEKIDVDEKGVISGLF    561
                      *** ********* :* *:***
```

Supplementary Fig. S1. Comparison of the Fhs amino acid sequences of *T. acetatoxydans* and *A. woodii*. The two different Fhs proteins of *T. acetatoxydans* are labeled as 1 and 2. Sequence alignments were done by ClustalOmega multiple sequence alignment.

```
A. woodii               MEFDAVGTEQSSISEKKCTEFVEALYSKAAVPGGGGAAALVGAVGTALAGMVGNLTTGKK    60
T. acetatoxydans 1      -----------MLIEKSCKDFVEVLSSKEPVPGGGGAAALVGAIGMALGNMVGNLTVGKK    49
T. acetatoxydans 2      ----------MKLADKTCNEFVNELASKNPVPGGGGAAALVGAIGVALSSMVCNLTTGKK    50
                                    : :*.*.:**: * **  *************:* **..** ***.***

A. woodii               KYAAFEDDIQRILKEAQILQDRLLAMIDEDAKNFLPLSKAYGLPKETEAEKAYKEKTLEE    120
T. acetatoxydans 1      KYKDVESEVYAIMEKATKLQEALLSMVDGDAEVFGEVAKVYSMPKETHKEKEARAEAMEK    109
T. acetatoxydans 2      KYAQFEDDIQDILKKAQELQNSLMQMIDEDAENFLPLSKAYGMPKGTEQERKLKEETLEK    110
                        **  .*.::  *:::*  **: *: *:* **: *  ::*.*.:** *. *:  : :::*:

A. woodii               CTKVACSIPLEIVEVCYKAVLLQEELVGKGSALAISDVACGVQCLRAAMISGWVNVLINI    180
T. acetatoxydans 1      ALKQACTVPMDIIRTAVEAIKLQARLSEIGSIMALSDVGVGVLCLKTALLSGKLNVIINL    169
T. acetatoxydans 2      ALKQACEVPIKIVKACYDAIKLHAELVDKGSRLAISDVGVGVQCLRAAIISGQLNIVINI    170
                        . * ** :*:.*:... .*: *: .*   ** :*:***. ** **::*::** :*::**:

A. woodii               KTIKDKDYVDDVNKRVKPMLEKGVEICDRVYADVEKQLL-    219
T. acetatoxydans 1      NGIKDEDFVLKISKEMDALVEEGVKIADETYDKVEQRIKK    209
T. acetatoxydans 2      NSIKDSVYVDRIKKEVMPLVKEGVNLADKVYEKVEQILSR    210
                        : ***. :*  :.*.:  ::::**::.*..* .**: :
```

Supplementary Fig. S2. Comparison of the FchA amino acid sequences of *T. acetatoxydans* and *A. woodii*. The two different FchA proteins of *T. acetatoxydans* are labeled as 1 and 2. Sequence alignments were done by ClustalOmega multiple sequence alignment.

```
A. woodii            MAAKLLSGKEVSESMLAEVLKDANELKAKGITVKMAIMRVGEDPGSISYEKSIITRMGKS    60
T. acetatoxydans 1   -MAKILSGKEVAAALKDKLTEEVKKLKEQGKALGLCIVMAGDRPDSASYIKGAVKRCTDV    59
T. acetatoxydans 2   -MGILMKGKPVADAIKEELKAEVQDLKAKGIIPKLTIVRVGADPGDMSYERGALKAMANI    59
                       . ::.** *: ::  ::  :.:.** :*     : *: .*  *.. ** :. :.   .

A. woodii            NIEVESVQFPIDVTEADFIAKLQSINEDKNIHSVLIFQPLPDQIDAEKIKYLLSPEKDPD    120
T. acetatoxydans 1   GIDCQVEKLPEEISQDEFLKVLDRLNKDSKINGIIIMRPLPRQISEDVVKYKIAPEKDVD    119
T. acetatoxydans 2   NIDTDARALPENIEQTDFISELEKINADTSTHGILILRPLPKQLDEKVIKNFIAPEKDVD    119
                     .*: :   :* :: : :*:  *: :* *.. :.::*::*** *:. . :*  ::**** *

A. woodii            ALNPTNLGKLMIADERGFFPCTAEGVMEMFKFYNIDVKGKDVVVINNSNVLGKPLTIMLT    180
T. acetatoxydans 1   CFSPINVSKVMSGDNDGFAPCTPAAVIEILDYYGIGLEGKRAVLIGRSMVVGRPLAMMLL    179
T. acetatoxydans 2   CFNPINVAKVFEGDETGFPPCTPSAVMEILKFYNIELMGKNAVVIGRSMIVGKPAAMLLL    179
                     .:.* *:.*:: .*: ** ***  .*:*::.:*.* : ** .*:*..* ::*:* :::*

A. woodii            NEFATVTMCHVFTKDTASYTKKADIVVTACGIYGLVKPDMLSEDCILIDVAMAQMKDENK    240
T. acetatoxydans 1   KRNATVTICHTRTKNLPQETQRAEILIAAAGKARMVTKDMVSEGAVVIDVGINFD-----    234
T. acetatoxydans 2   KEHATVTVCHSRTRDLPLVASGADILVVGIGRAKMVNSNYIKDGAVVIDVGINMD-----    234
                     :. ****:**  *::    :. *:*::.. *   :*. : :.:..::***.:

A. woodii            EFVLNEEGKKIRTGDAH-VDCLNKVAMITSATPGCGGGTGPITTALLAKHVIKACKMQNG    299
T. acetatoxydans 1   -----ENGKM--CGDVDFDSVSPIAGQIT----PVPGGVGSVTSTVLAKHVIEAFKLQQG    283
T. acetatoxydans 2   -----EDGRL--CGDVDTDNCINKASMIT----PVPGGVGSVTTSVLAKHVVQACKMQLS    283
                          *:*:    **..  .    .. **       **.* :*:::*****::* *:* .

A. woodii            LL     301
T. acetatoxydans 1   L-     284
T. acetatoxydans 2   LL     285
                     *
```

Supplementary Fig. S3. Comparison of the FolD amino acid sequences of *T. acetatoxydans* and *A. woodii*. The two different FolD proteins of *T. acetatoxydans* are labeled as 1 and 2. Sequence alignments were done by ClustalOmega multiple sequence alignment.

```
A. woodii           MLITQLKSKESIESLA--VGKVVIINCHGCKEVYFPEHQA--DELQKELSD-NKEVIQII    55
T. acetatoxydans 1  MIISKQKPFEEILEFLNGKNKIFITGCSLCATSCMTGGQQQIEER-KNLRNKGK-LALFT    58
T. acetatoxydans 2  MIISQNKPFEEITEYLKDCEKIVLIGCAQCATACKSGGEEELIAMKKRLEEIGKQVLAFI    60
                    *:*:: *  *.* .      *:.: .*  *        :       *.* : .* :  :

A. woodii           TTDYICNPDNLKLQLKKHMDKINLADTILVFSCGVGIQTVAAQFEDKKVYSCCDTYQLPG    115
T. acetatoxydans 1  HPAISCKKQLKNKNLKLTRRMPSY-----VWLVV-TAPRPWQI-AKLRVYPANDTLFIGE    111
T. acetatoxydans 2  VPETSCNYLLIRRDLKKIKDEINEADAILSFACGDGTQTIAKQ-VKIPVYPGNDTLFVGE    119
                         *:    . :**      .       :              .  **   **  :

A. woodii           FQGVTPLNVDCGQCGECYLNGTGGICPITACSKSLINGQCGGAKNGMCEVDKDMECGWER    175
T. acetatoxydans 1  IERIGQYQEACKACGQCELGWTAGICPITKCAKSLLNGPCGGAKNGKCEINPENDCAWIL    171
T. acetatoxydans 2  VERVGQYSEACRTCGECVLGLTGGICPVTRCAKSLLNGPCGGAKDGKCEVNPENDCAWIL    179
                    .: :   .  *  **:* *. *.****:* *:***:** *****:* **:: : :*.*

A. woodii           IYRKLEKLNKLDNMKYDAPKVRNFGNSKPE-------------  205
T. acetatoxydans 1  IYNRLKDLDMLNTLLEIKPPKDYSKSLHPRKLAASK-------  207
T. acetatoxydans 2  IYKRLKELGQVENLIQIQQPKDYSKTTYPRNLNLREKDGGGRQ  222
                    **.:*:.*. ::.:                 .  *.
```

Supplementary Fig. S4. Comparison of the MetV amino acid sequences of *T. acetatoxydans* and *A. woodii*. The two different MetV proteins of *T. acetatoxydans* are labeled as 1 and 2. Sequence alignments were done by ClustalOmega multiple sequence alignment.

```
A. woodii             -MRITELFQRDEFVLTAEVGPPKGVELDHLIKDAHEYLKTRVHALNVTDNQSSVMRTGSL    59
T. acetatoxydans 1    -MGLKEKLEHNEFAITAEMAPPKGIDFGRIRECA-QAVKGRVDAVNVTDFQSAVVRSSSL    58
T. acetatoxydans 2    MSLLQKALESGEFAVTAELAPPKGTDFSHALQNA-ELFGDRVHAINVTDFQSSSLKATSL    59
                         : : :: .**.:***:.**** ::.:  : * : .  **.*:**** **: ::: **

A. woodii             ATCKVLKDEGLLPIFQITCRDRNRIALQSDVLSAALLGIENLLLLTGDYTTLGDHPQAKP    119
T. acetatoxydans 1    GGAKVLLEEGLEPVMQITGRDRNRIAIQGELLSAAALGIKNILALTGDHPSIGDHPEARG    118
T. acetatoxydans 2    AMCKALIDLGLEPVFQITGRDRNRIAIQGEMLSAGFFGVKNLLALTGDHTTVGDNPGAKP    119
                      . .*.* : ** *::*** *******:*.::***. :*::*:* ****: ::**:* *:

A. woodii             VFDLDSVSLIHAVKRLEEGFDLAGNAVDGAPPKFAKGAVVSPCSDSVDVQLAKMEAKVKA    179
T. acetatoxydans 1    VFDMDSVGILQAATKLMNGEDFNGHELSGIPE-YFLGASVTPLYEPVELQIIKMVKKIQA    177
T. acetatoxydans 2    VYDLDSIGILQAATMLASGVDMGGNKLEGSPS-FFLGASVTPEYDPIELQLIKMKKKIIA    178
                      *:*:**:.:::*.. * .* *: *: :.* *  :  ** *:*  : :::*: **  *: *

A. woodii             GCEYFQTQGVYEPEKFIKFMEQAKQFGVPVQLGLIIPKNVGMCRYMNANVAGVHVPDDMI    239
T. acetatoxydans 1    GARFFQTQAVYDIDVIKGFMDKIKDLDAKVLVGVIPLKSAGMAKHMNNNVPGIHVPDEII    237
T. acetatoxydans 2    GAKFFQTQAVYDIRTMEKFKEITKHLNTKILVGIIPLKSAGMARFMNKKIPGINVPDELI    238
                      *..:****.**:   :  * :  *.:.. : :*:*  *..**.:.** :: *::***::*

A. woodii             EELKADKEKTKAGDTGVEICARLIREC--KDYCQGVHIMSLGWENRIPEILDMAGIAPIA    297
T. acetatoxydans 1    DKMKKAQDKEKE---GLNIAAEFIKQVREEKICAGVHIMAVGAEENLPVLFDMCGF----    290
T. acetatoxydans 2    DRLKLSKDPVQE---GIKIAGEFIVELKNRNLCDGVHIMAIGAEENVPLILDAAEL----    291
                      :.:*  ::  :    *::*...:* :   .. * *****::* *:.:* ::* . :

A. woodii             K       298
T. acetatoxydans 1    -       290
T. acetatoxydans 2    -       291
```

Supplementary Fig. S5. Comparison of the MetF amino acid sequences of *T. acetatoxydans* and *A. woodii*. The two different MetF proteins of *T. acetatoxydans* are labeled as 1 and 2. Sequence alignments were done by ClustalOmega multiple sequence alignment.

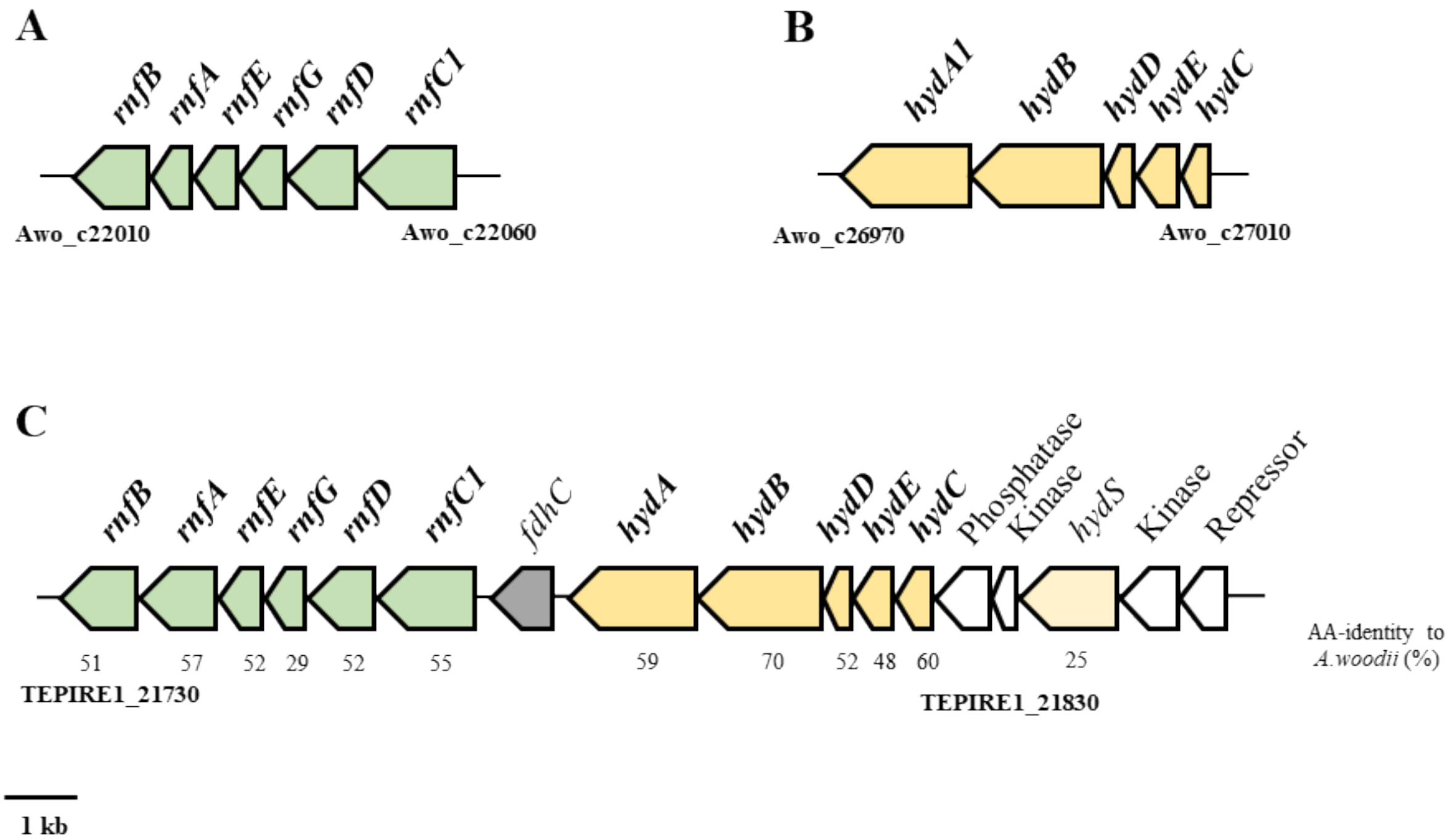

Supplementary Fig. S6. Genetic organization of the Rnf-complex and electron bifurcating hydrogenase encoding genes of *T. acetatoxydans* in comparison to *A. woodii*. The genome of *A. woodii* harbors two separated gene cluster encoding the Rnf-complex (*rnfB*, *rnfA*, *rnfG*, *rnfD*, *rnfC1*) (A) and the electron bifurcating hydrogenase (*hydA1*, *hydB*, *hydD*, *hydE*, *hydC*) (B), while in the genome of *T. acetatoxydans* the Rnf-complex and electron bifurcating hydrogenase encoding genes are located in one gene cluster (C) separated by one gene (*fdhC*), encoding a formate transporter. Additionally, the gene cluster of *T. acetatoxydans* harbors genes encoding a hydrogenase HydS, a phosphatase and kinase, forming a postulated sensoric hydrogenase. The gene amino acid identities (%) of the comparison of the correlating gene products are indicated by numbers.

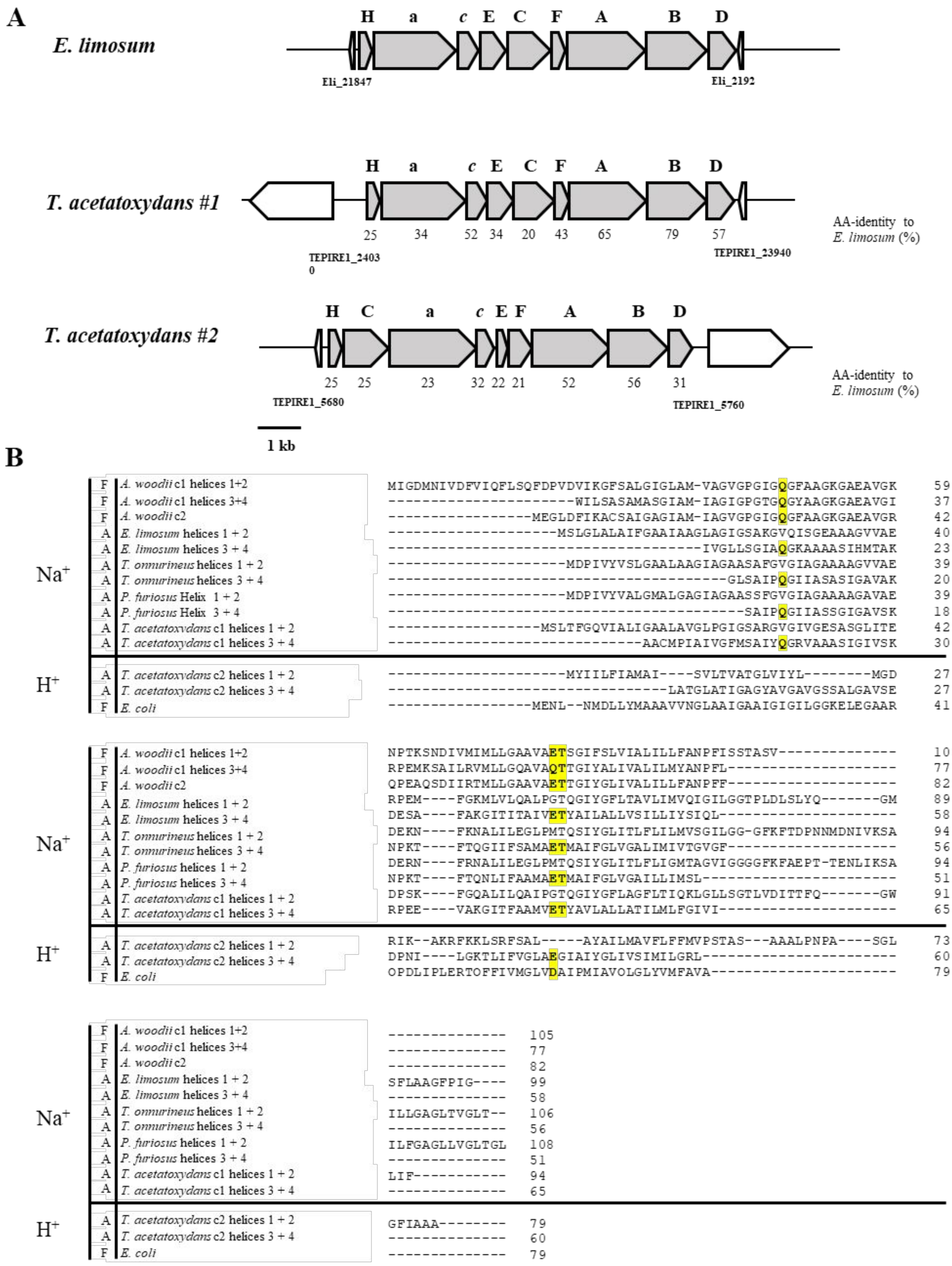

Supplementary Fig. S7. Comparison of the genetic organization of the A_1A_O ATP synthase encoding gene cluster of *T. acetatoxydans* and *E. limosum* (A) and comparison of the ATP synthase *c*-subunits (B). The *c*-subunits of the two A_1A_O ATP synthases of *T. acetatoxydans* were compared to *c*-subunits of Na^+-dependent (*A. woodii*, *E. limosum*, *Thermococcus onnurineus*, *Pyrucoccus furiosus*) as well as H^+-dependent (*Escherichica coli*) ATP synthases of the F_1F_O- and A_1A_O-type. The amino acid sequences of *c*-Subunits consisting of four transmembranehelices were halved for better comparison. Sequence alignments were done by ClustalOmega multiple sequence alignment.

```
Na+  A. woodii           --MNFMKNLTRGIIRENPTFVLVLGMCPTLAVTTSAINGMGMGLATMLVLIGSNVAISAL  58
     E. callanderi       --MGSFKHFTNGLIKENPTFRLLLGMCPTLAVTTSAINGLGMGLATTVVLIGSNVVISAL  58
     T. maritima         --MSRLRELTKGIIKENPTYVQVLGMCPTLAVTTSAINGLGMGLATTAVLTMSNVVISLI  58
     B. fragilis         --MNNFKVLMNGIVKENPTFVLLLGMCPTLGTTSSAINGMGMGLATMFVLICSNVVISLI  58
     T. acetatoxydans    --MQLFKDFINGIWNENPIFRMVIGICSALAVTTAANNGIAMGMALTFVLTCSSILISLL  58
H+   S. ovata            --MNYWEIFKKGIFEMNPIFRLALSLCPALAVTATVFNALGMGFAVMAVITANNIVVSLV  58
     C. ljungdahlii      -MKNLWNIFKKGLIAENPIFVLALSLCPALATTSTAINGFTMGLCVLFVITCNNTVVSII  59
     C. autoethanogenum  -MKNLWNIFKKGLIAENPIFVLALSLCPALATTSTAVNGFTMGICVLFVITCNNTVVSII  59
     R. capsulatus       MSENYAKITRDGLWDKNIVTGQMLALCPALAITGTATNGLGMGLATTAVLIVSNVAISAL  60
                               .    *:   *      :.:* :*. * :. *.: **:.   *:  ..  :* :

Na+  A. woodii           RKVIPDNIRIPAFVVVIASFVTIVGMLMKAYVPALDAALGIFIPLIVVNCIILARAEAFA  118
     E. callanderi       RKIIPDKIRIPAFVVIIASFVTIVGMLMKAYVPALDAALGIYIPLIVVNCIILGRAEAFA  118
     T. maritima         RKIVPDKIRIPIFIVVIASFVTMIDLLMHGFAYDLWKTLGLFIPLIVVNCIIMGRAESFA  118
     B. fragilis         KNLIPDMIRIPSFIVVIASFVTLLQMVMQAYVPGLYATLGLFIPLIVVNCIVLGRAEAFA  118
     T. acetatoxydans    RNIIPAKVRIPCYIIVVATFTTVVDLFMKAYFPSLYEVMGIFIPLIVVNCIVLARAEAFA  118
H+   S. ovata            KKLVNPKVRVPMFITIIATIVTLTILVLEAYAPAVYKELGLYLELVVVFAIILARAEVFA  118
     C. ljungdahlii      KNVVNPKVRVPVYITCIATIVTVVELVMQAYAPLLYKQLGIYLALVVVFAIILARAETFA  119
     C. autoethanogenum  KNVVNPKVRVPVYITCIATIVTVVELVMQAYAPLLYKQLGIYLALVVVFAIILARAETFA  119
     R. capsulatus       RKVIAPEIRIPAFILIIAAIVTVVDLALNAFLHDLHKVLGLFIALIVTNCAILGRAEAFA  120
                         ::::   :*:* ::  :*::.*:  : :..:   :   :*::: *:*. . ::.*** **

Na+  A. woodii           FSNGIADSFADAVGMGLGFTLALTILGSIREILGAGSIFGFSLF-------------GAA  165
     E. callanderi       FSNPVLASFADGAGMGLGFTLSLTVLGSIREIIGAGAIFGLSIF-------------GAG  165
     T. maritima         SKHGVLDSMLDGLGVGLGFTGSLVLLGSVRELFGNGTIFGYKVW-------------ELK  165
     B. fragilis         AKNNALSSMFDGIGMGLGFTIALTLLGAVREFLGTGKIFNLSII-------------PEE  165
     T. acetatoxydans    SKVGPVRATADALGMGLGYTWAITLISMIRELMGAGTLFGYPIM-------------GEG  165
H+   S. ovata            MKNDVLPSAVDGLGMGAGFALAMLFIGAFREVFGTGTILSGTSMAM--------QVFPPS  170
     C. ljungdahlii      SKNPVVPSFFDGLGMGCGFTLALTIIGMIRELFGSGTIFGA-------------NVFGAS  166
     C. autoethanogenum  SKNPVVPSFFDGLGMGCGFTLALTIIGMIRELFGSGAIFGV-------------NVFGAS  166
     R. capsulatus       SRRGVLASAVDGLTMGVGFTLALIVIGALREIFGSGTLFAQASVLLGAQFSFMELTLFPD  180
                                :  *.  :* *:: :: .:. .**.:* * ::

Na+  A. woodii           YEPVLLMILPPGAFLTLGLLIGLINWKTKKA-----------------------------  196
     E. callanderi       YEPVLIFILPPGAFLTLGVGIAAINKITEKLEAK-----KSNKEDSLS------------  208
     T. maritima         ---IFLEILPPGAYITLGLLSALFTYIGIRRKKR-----GEAK-----------------  200
     B. fragilis         YG-MLVFVLAPGAFIALGYLIALINSLKKAN-----------------------------  195
     T. acetatoxydans    FTPWTIMIMPPGAFIVMGILVGLMNMITRKQVE---------------------------  198
H+   S. ovata            YDGPMIIILAPGAFLVIGLMIGMFNVIGEYQDKQAAKAKAASQLTMQRSEAV--------  222
     C. ljungdahlii      YNPALIMILPPGGFILIGYLVAIVKVYNQHMEKIKMQKLE----KANGGEA---------  213
     C. autoethanogenum  YNPALIMILPPGGFILIGYLVAIVKVYNQHMEKIKMQKLE----KANGGEA---------  213
     R. capsulatus       YPGFLILILPPGGFIVLGFLFAAKRLIDARKLTIEQEIKQMRKERVFTAAGVLKPQPEMG  240
                              : :: **.:: :*   .

Na+  A. woodii           ---  196
     E. callanderi       ---  208
     T. maritima         ---  200
     B. fragilis         ---  195
     T. acetatoxydans    ---  198
H+   S. ovata            ---  222
     C. ljungdahlii      ---  213
     C. autoethanogenum  ---  213
     R. capsulatus       EEA  243
```

Supplementary Fig. S8. Comparison of the RnfE subunits of *T. acetatoxydans* with RnfE subunits of other Rnf-complexes. The amino acids essential for binding of the coupling ion are highlighted in yellow. RnfE-subunits of Na^{+}-translocating (*A. woodii*, *E. callanderi*, *Thermotoga maritima*, *Bacteroides fragilis*) and H^{+}-translocating Rnf-complexes (*Sporomusa ovata, C. ljungdahlii, C. autoethanogenum, Rhodobacter capsulatus*) were used for the comparison to the RnfE subunit of *T. acetatoxydans*. Sequence alignments were performed by ClustalOmega multiple sequence alignment.

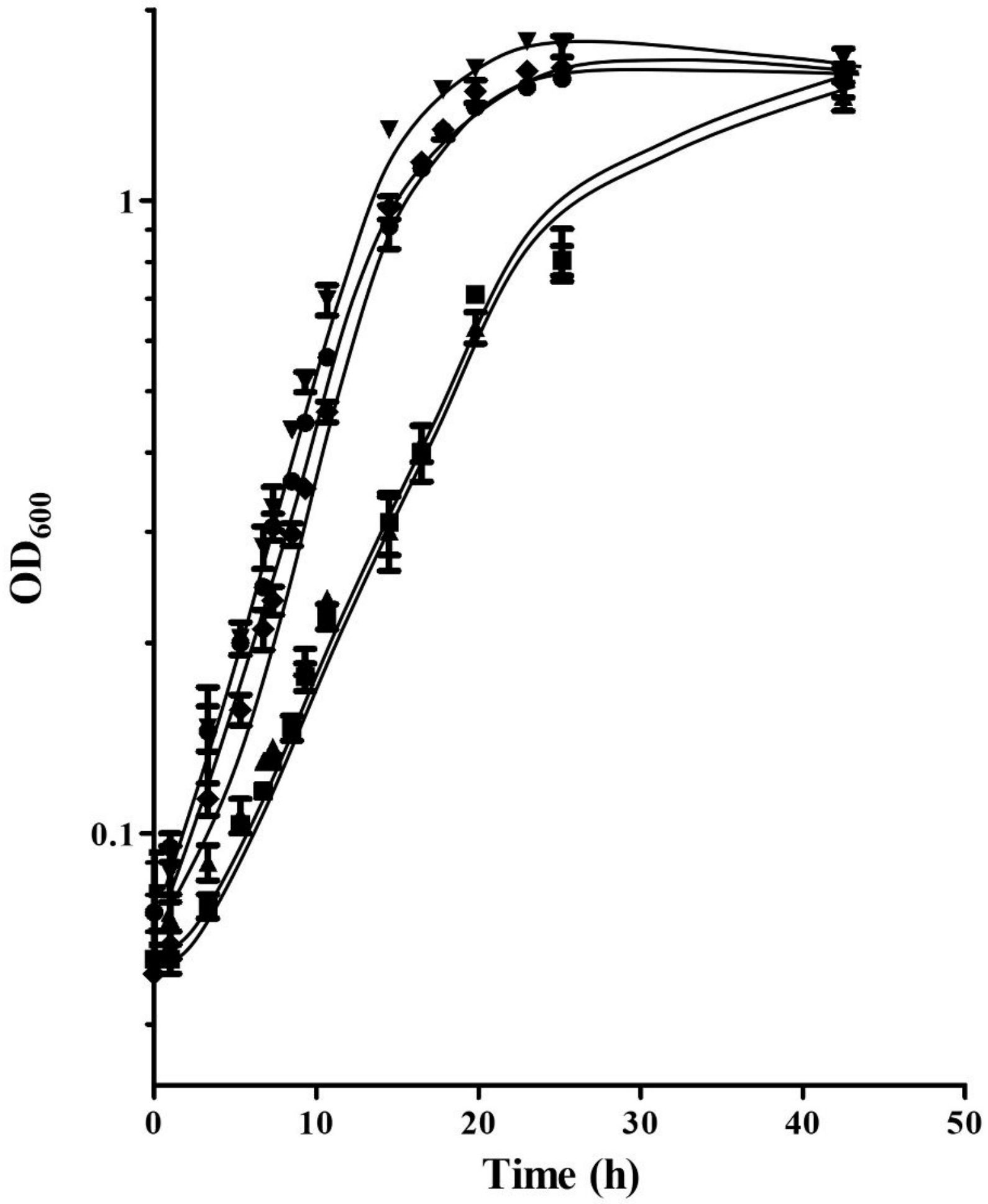

Supplementary Fig. S9. Growth of *T. acetatoxydans* with glucose and formate as carbon and energy sources is stimulated by Na^{+}. A preculture of *T. acetatoxydans* was grown in Na^{+}-limited medium (contaminating Na^{+}-concentration: 0.84 mM) at 45 °C with 20 mM glucose and 20 mM potassium-formate as carbon and energy sources to inoculate 5 ml Na^{+}-limited medium (5 %) with 20 mM glucose and 20 mM of sodium formate (●), potassium formate (■) and ammonium formate (▲). Additionally, *T. acetatoxydans* was grown in similar medium with 20 mM glucose and 20 mM potassium formate (♦) or 20 mM glucose and 20 mM ammonium formate (▼) in the presence of 20 mM NaCl at 45 °C. All data points mean ± SEM; *N* = 3 independent experiments.

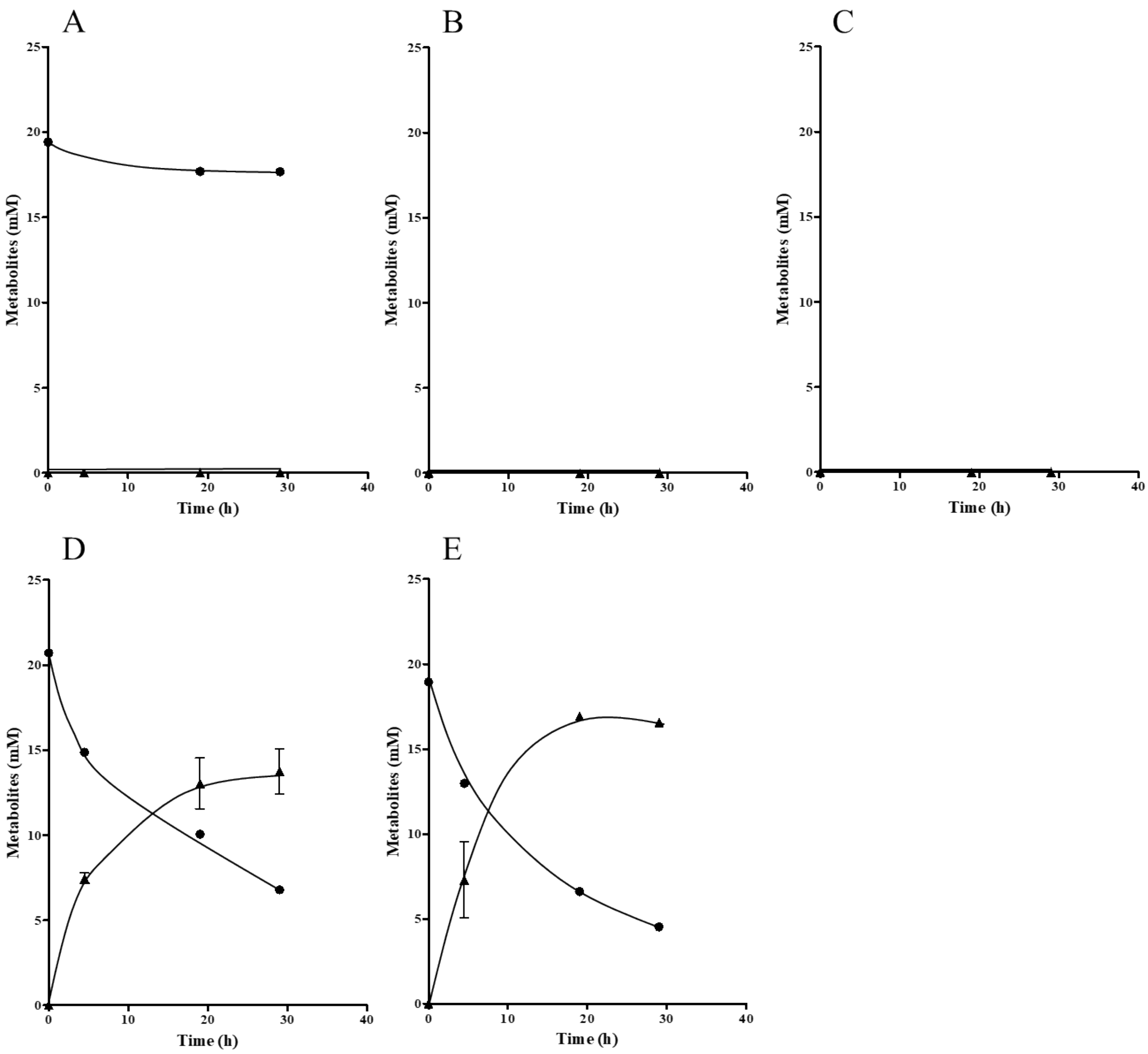

Supplementary Fig. S10. Acetogenesis from C1 compounds by resting cells of *T. acetatoxydans*. *T. acetatoxydans* was grown in CO_2/$KHCO_3$-buffered medium at 45 °C with 20 mM glucose and 20 mM formate as carbon and energy source to late exponential growth phase, harvested, washed and resuspended to a final protein concentration of 1 mg ml^{-1} in 10 ml imidazole buffer (50 mM imidazole, 60 mM $KHCO_3$, 20 mM KCl, 20 mM NaCl, 20 mM $MgSO_4$, 2 mM DTE, 4.4 µM resazurin, pH 7.0) in 115 ml serum flasks. If not otherwise stated, cells were incubated under a N_2/CO_2 atmosphere (80/20% [v/v]) at 45 °C. As substrate 20 mM formate (A), 1 bar (100 %) H_2 + CO_2 (B), 20% CO (C), 20 mM formate with H_2 + CO_2 (1 bar, 100 %) (D) or formate with 20 % CO (E) were used. The concentrations of acetate (▲) and formate (●) were determined at each point. All data points mean ± SEM; N = 2 independent experiments.

```
T. acetatoxydans MT1 - 1      --MTTTSAQEFAKERLARVEAAINLKEPDRVPFSG-LGGDIIPVYAGITHHEFCYDY--E      55
T. acetatoxydans MT1 - 2      MEMTTKSAQELAKERLDRMAAALNLEEPDKVPLRG-FGGDIIPAYAGITQKEFCYDY--E      57
T. acetatoxydans MT1 - 3      ----MSDVKKLQQERINIYQDIFDNKIPKRVPVNVSLPLEVIAQYGKIDLVEAQW--NLE      54
T. acetatoxydans MT1 - 4      -----MNAQQLKEYRTQLYKDVYSGIIPDRFPVHDGLSAEYLIEYAGKDFLTTQYQYTTE      55
T. acetatoxydans MT1 - 5      ----MANIKDVQRKRAQLFHDLSDGKIPERVPVGGNIGIEFCIQYSGLPLAKTQW--TLE      54
T. acetatoxydans MT1 - 6      MEMTTKSAQELAKERLDRIEAAINLEEPARVPCWG-IGGEIVASYSGFTQYDLAYDP--E      57
M. shengliensis MotB1         -----MDVEELRKERLERLERALKCKEPDRVPILV-FNDVWCGTYSGYTAKEVYFEY--D      52
M. shengliensis MotB2         -----MDIEELRRERLERLEAAIKCKEPDRVPIRA-FTHIWHGAYAGHTAKEVLFDN--G      52
                                    . :.. . *        .   * :.*    :       *.        :

T. acetatoxydans MT1 - 1      KTRRALAKFNKDFPCDWTFAAGFTGVGVPPLTYAFAE-YPDLSTVFG-MFGTIHDILNDK      113
T. acetatoxydans MT1 - 2      KSLKAIEKFLKDFPFDSPG-GSIPGLDGRAFSIAFAE-FDDIAPNTTFITGPMHDALGDK      115
T. acetatoxydans MT1 - 3      KLEESADKICQTVYSDTCV---FMGTFRLPSFYQILKSQSFVMALNG-------------      98
T. acetatoxydans MT1 - 4      ILEEIGEKAMELVRGDS-F---SLAWVRNPISLMFQKSKAFVMSKTG-------------      98
T. acetatoxydans MT1 - 5      NIEEAMDKACQITGSDL-Y---PLGPARPPAPYQILGSRTNVMGSKG-------------      97
T. acetatoxydans MT1 - 6      KSLKAIEKFLKDFPFDTSM-AGLAGLDGRVFSIAFAE-HPDISPAVTFITGPLHDIWGDK      115
M. shengliensis MotB1         KWIKATLNVAKDFEFDYIF--TLNGLEGMFLFASFIEKAPEVATNMRFVTAPYHDILQDV      110
M. shengliensis MotB2         KCKDAWLKVAKDFDFDTFT--VISGLAGWIYAVALLD-QQDISATAPLILGPTHIALMDV      109
                                     :  :    *        .         :      :

T. acetatoxydans MT1 - 1      CTRYPGRELEENCSPQFVGGTFMEPDEYDELIKDAVGFIAETVLPRICRNLET---PIKA      170
T. acetatoxydans MT1 - 2      YYRFPGREIDEDATPQFLGGTFMEPDEYDKLIENPVKFIAETILPRVCRNLET---PRQA      172
T. acetatoxydans MT1 - 3      -------------FIQHPEVTGMLPEDYDYLIEKPYDCIMERVLPRQYKALDYNNDPINS      145
T. acetatoxydans MT1 - 4      -------------FFQHPEVSGFEPEEYDEFIRNPFDFMVEKIAPRLNPALD--TDPISR      143
T. acetatoxydans MT1 - 5      -------------FIQHPEVSVMEADEYDEFIKNPHEFIMEKALPRMNKELD--TDPVTR      142
T. acetatoxydans MT1 - 6      YTRFPGREIDVNATPQFVGGTFMEPDEYDKLIENPVKFVAETILPRVCRNLET---PRQA      172
M. shengliensis MotB1         YTRWPGRELREDMHPQFIGKEIMKKEEYNKFIEAPIDFMHEVALPRICKNVAN---PGSA      167
M. shengliensis MotB2         YTRWPGYELEENAHPQFIGKEIMKVDEYDQLIENPLEFINKVAMPRINKKLSN---VGSA      166
                                             *.     :  ::*: :*.     : :   **    :

T. acetatoxydans MT1 - 1      ----MATWVRMGMEAEKMAAFMQGVNEDMIKLGIPPIP---TTLGMAPLDLIGDGLRDVT      223
T. acetatoxydans MT1 - 2      ----MATWVRLGMEFDRFSAAGRQLGAISAQLGYPRFP---MGGAYAPLDIIGDFLRGIS      225
T. acetatoxydans MT1 - 3      AISLTKSMLAYN---NDFMQIGMLIGKLTEKYGYYPGSLSGGGFTEAPFDFLADQLRGFR      202
T. acetatoxydans MT1 - 4      SVIFASAVLAQM---EHQRVFDVANNMLAEKYGFFIAPPGTVGLQLIPFDFLADFSRGFT      200
T. acetatoxydans MT1 - 5      SIVYAKAVKAFF---DYTDVYAQIDAKLIEKYGFYKIPLESAGMALSPLDFIADYLRGFK      199
T. acetatoxydans MT1 - 6      ----MATFVRVGMEVAGSASTMGKLAEISAKLGYPDFP---MGWGYAPLDIIGDFLRGVG      225
M. shengliensis MotB1         --DYNAALAKLGMEAGKYNAATNSFIMELMKIGYPMWP---MAHGYAPLDFIGDFLRDIK      222
M. shengliensis MotB2         --EYNAALAKYGVELARFGAFMAEVSMELAKIGYPAIP---MSWGYAPLDFIGDYLRDIK      221
                                    :                       : *              *:*::.*  *..

T. acetatoxydans MT1 - 1      NVVLDIHRCPEKVKKACEALIEPILKVGLAL--------KPFG--VKIAFIPLHLNEYLS      273
T. acetatoxydans MT1 - 2      NVVLDIRRYPDKVKKAAEALVDPIVNYGLSY--------KKMG--FDLVMIPLHLNEYLS      275
T. acetatoxydans MT1 - 3      GISMDIRRMPEKVAEACEALYPIAFKKGLPSNP----------SKYAPVFIPLHMPTFMR      252
T. acetatoxydans MT1 - 4      KIVLDIKRYPEKVLEALEALVPYAIWLGRTPKT----------SILGANMIMTHMATFLN      250
T. acetatoxydans MT1 - 5      GIIMDMRRYPEKVAEACEALMPLILKMGTPPMP----------SKYGQTFMPLHMAPFLR      249
T. acetatoxydans MT1 - 6      TVVLDIHRYPDKVKKATEALLEPVTNYALTS--------KQMG--AKYAMFPLHLNEYLS      275
M. shengliensis MotB1         NIVIDLYRVPDVVIQAVDAITPHLVKTASISGSVPEEVMKMLGVNLIWCFYPLHLNEYLN      282
M. shengliensis MotB2         NMVMDLYRHGDVVKQAVEAVTPLLIKVAEVSAPPAEVRKQIFGTDVVECLIPLHLNEYLN      281
                               : :*: *  : * :* :*:       .                     :   *:  ::

T. acetatoxydans MT1 - 1      PKLYNEFYWPYLKEVINGFAKEGIKSMVFFEGRHDAHLETILELPAGWGIAYFEKTDVRK      333
T. acetatoxydans MT1 - 2      PKLYKEFYWPTLKEVILRLGKEGIRSWVFFEGHHEQHLETILELPKGWGAAYFEKTDVVK      335
T. acetatoxydans MT1 - 3      EKDFEKLFWPTFKRMVDEYASIGVHCNLFCEDDWMRYLDYLYELPTDTV-LMFEYGDPKL      311
T. acetatoxydans MT1 - 4      RKDFEKFYWPTFYKLCHICAERGQSMSIFCEDDWTRYIEYLQELPQGTR-LLMEYGDPQK      309
T. acetatoxydans MT1 - 5      TSDFEKIYWPTFSRTIQAFADRGQTVFMFCDHDWMRYLDHLYELPENTW-MAFEFGDPKV      308
T. acetatoxydans MT1 - 6      PKLYNEFYWPTLKSIILRLYEEGIKSIVFFEGHHEQHLETILELPRGWGVAYFEKTDVIK      335
M. shengliensis MotB1         PKQYREYYWPSLLHCWKENVKAGLLPFVLFEGRHDAHLETLLEIPKRGIAGMFEKTDGRK      342
M. shengliensis MotB2         PKLYNEFFWPSLNEVIKEVVKMGQTPFVLFEGRHDAHLETLLEAPRRKVVGVFEKTDPRK      341
                               . :.: :** :        . *    :: :     ::: : * *       :*  *

T. acetatoxydans MT1 - 1      AKKVLQGHTCVMGGVPISLIISGTPEKIDAYVKNLLEEVKPGGGYILTTSVG--TAPRET      391
T. acetatoxydans MT1 - 2      AKEVLQGHTCVVGGIPISLIVTGTPAKIDEYIKSLLEQVKPGGGFMLSTSIG--SAPADT      393
T. acetatoxydans MT1 - 3      IKDKLGKKHIITGLYPIMNLKTKTAKECVDELRKYIDILAPGGKYVFGFDKN-PLLLDDI      370
T. acetatoxydans MT1 - 4      FKDKLGKKMVLSGFYPVSLLKTGTKQQCIDKAKELIDILAPGGNYIFGFDKQ-ALRLSDI      368
T. acetatoxydans MT1 - 5      VKEKLGKRHVVAGLYPMTYLKTGTKQQCIDKAKELLDIMAPGGRYYFDFDKS-LISIDSA      367
T. acetatoxydans MT1 - 6      AKEVLKDHTCVMGGIPISLIVSGNPEQIDEYIKNLLEQVKPGGGFILAPGVG--AAPAET      393
M. shengliensis MotB1         VKELVGNDMCIMYGPTNTLLASATPEKVYEWTKELVLDMKEGGGFVLFPGVDAAGISREA      402
M. shengliensis MotB2         VREVLGDHVILVSGPPNSLLIGGTPQKVEDFMKKLLEDCKEG-GMMIYPGVD-GGISRDA      399
                              :. :     :        :   .  :     :. :     *    :  .        .

T. acetatoxydans MT1 - 1      PIENMHALINAVEKYGKY------------------------------------------      409
T. acetatoxydans MT1 - 2      PLENITALIEAVEKYG--------------------------------------------      409
T. acetatoxydans MT1 - 3      NLESLCAVAETVRDYGVYSNPGEVAGMEFN--KEDYKVESSRKIESKYYKTWEQYKALNP      428
T. acetatoxydans MT1 - 4      NPENYVAVMEYVLDNSKYTNAGQPVTTAKREDSIVKFSHTYPEFKSKYIVSFDEFIEDYP      428
T. acetatoxydans MT1 - 5      NIENYTAVTRYMAENSYYNNAGAAAASDGNAGRKFIDVGEIRPFRSKYYKTWDEYKAKHP      427
T. acetatoxydans MT1 - 6      PPENITAVIEAVEKYG--------------------------------------------      409
M. shengliensis MotB1         KLENIKAMIEAVHKYGQY------------------------------------------      420
M. shengliensis MotB2         KPENVKAMIEAVEKYGTY------------------------------------------      417
                                *.  *: . : . .

T. acetatoxydans MT1 - 1      ------------------------------      409
T. acetatoxydans MT1 - 2      ------------------------------      409
T. acetatoxydans MT1 - 3      EV----SDFAKTKLQGFEEALFQYLMYLLL      454
T. acetatoxydans MT1 - 4      PADDRAEPLMRAAYDKYTSMVVPSLGGVF-      457
T. acetatoxydans MT1 - 5      EINPELESIIAEKLQVYEEMLFMFF-----      452
T. acetatoxydans MT1 - 6      ------------------------------      409
M. shengliensis MotB1         ------------------------------      420
M. shengliensis MotB2         ------------------------------      417
```

Supplementary Fig. S11. Comparison of the amino acid sequences of the MTI methyltransferases of *T. acetatoxydans* to the methyltransferases MotB1 and MotB2 of *M. shengliensis*. The six different MTI proteins of *T. acetatoxydans* are labeled (1, TEPIRE1_3210; 2, TEPIRE1_3240; 3, TEPIRE1_3290; 4, TEPIRE1_3310; 5, TEPIRE1_3320; 6, TEPIRE1_3360). Sequence alignments were performed by ClustalOmega multiple sequence alignment.

Supplementary Tab. S1. Aminoacid identities of MT1 enzymes of *T. acetatoxydans* in comparison to the methyltransferases MtoB1 and MtoB2 of *M. shengliensis*.

**Locus-Tag**	**Amino acid identity to MtoB1 (%)**	**Amino acid identity to MtoB2 (%)**
TEPIRE1_3210	40	44
TEPIRE1_3240	42	44
TEPIRE1_3290	25	28
TEPIRE1_3310	28	28
TEPIRE1_3320	28	32
TEPIRE1_3360	42	43
